# Stratifying TAD boundaries pinpoints focal genomic regions of regulation, damage, and repair

**DOI:** 10.1093/bib/bbae306

**Published:** 2024-06-27

**Authors:** Bijia Chen, Chao Ren, Zhangyi Ouyang, Jingxuan Xu, Kang Xu, Yaru Li, Hejiang Guo, Xuemei Bai, Mengge Tian, Xiang Xu, Yuyang Wang, Hao Li, Xiaochen Bo, Hebing Chen

**Affiliations:** Academy of Military Medical Sciences, Beijing 100850, China; Academy of Military Medical Sciences, Beijing 100850, China; Academy of Military Medical Sciences, Beijing 100850, China; Key Laboratory of Carcinogenesis and Translational Research (Ministry of Education/Beijing), Department of Gastrointestinal Surgery, Peking University Cancer Hospital & Institute, Beijing 100142, China; School of Software, Shandong University, Jinan 250101, China; Academy of Military Medical Sciences, Beijing 100850, China; Academy of Military Medical Sciences, Beijing 100850, China; Academy of Military Medical Sciences, Beijing 100850, China; The First Affiliated Hospital of Harbin Medical University, Harbin 150001, China; Academy of Military Medical Sciences, Beijing 100850, China; College of Computer and Data Science, Fuzhou University, Fuzhou 350108, China; Academy of Military Medical Sciences, Beijing 100850, China; Academy of Military Medical Sciences, Beijing 100850, China; Academy of Military Medical Sciences, Beijing 100850, China

**Keywords:** 3D chromatin organization, topologically associating domains (TADs), TAD boundary, gene regulation, DNA double-strand breaks (DSBs), genomic stability

## Abstract

Advances in chromatin mapping have exposed the complex chromatin hierarchical organization in mammals, including topologically associating domains (TADs) and their substructures, yet the functional implications of this hierarchy in gene regulation and disease progression are not fully elucidated. Our study delves into the phenomenon of shared TAD boundaries, which are pivotal in maintaining the hierarchical chromatin structure and regulating gene activity. By integrating high-resolution Hi-C data, chromatin accessibility, and DNA double-strand breaks (DSBs) data from various cell lines, we systematically explore the complex regulatory landscape at high-level TAD boundaries. Our findings indicate that these boundaries are not only key architectural elements but also vibrant hubs, enriched with functionally crucial genes and complex transcription factor binding site–clustered regions. Moreover, they exhibit a pronounced enrichment of DSBs, suggesting a nuanced interplay between transcriptional regulation and genomic stability. Our research provides novel insights into the intricate relationship between the 3D genome structure, gene regulation, and DNA repair mechanisms, highlighting the role of shared TAD boundaries in maintaining genomic integrity and resilience against perturbations. The implications of our findings extend to understanding the complexities of genomic diseases and open new avenues for therapeutic interventions targeting the structural and functional integrity of TAD boundaries.

## Introduction

The enigmatic 3D structure of chromatin stands as a pivotal hallmark of eukaryotic genomes nestled within the nucleus, intimately linked to cellular biological functions and disease pathogenesis and progression [1, 2]. Recent advancements in chromatin conformation capture technologies have unveiled the hierarchical organization of mammalian chromatin into structural domains [3–6], notably the topologically associating domains (TADs) [7–9].

TADs manifest as local contact domain structures along the diagonal in Hi-C contact frequency maps [10], with TAD boundaries functioning as insulator elements to restrict genomic interactions. Initial methodologies for TAD delineation, such as the Directionality Index [7] and Insulation Score [11], segmented the genome into discrete bins, enabling the identification of TAD boundaries through the strategic interpretation of these indices. Yet, as the narrative of research progressed, novel methodologies have been developed for more nuanced identification and alignment of TAD hierarchy, including sub-TADs and nested TADs [10, 12]. The recently introduced OnTAD [13] algorithm adeptly identifies candidate boundaries by implementing an adaptive local minimum search technique across a spectrum of window sizes, performing exceptionally well and elucidating a multitude of biological phenomena intimately linked to TAD hierarchy. Despite these advancements, a degree of inconsistency lingers in the realm of TAD and sub-TAD identification, while the delineation of TAD boundaries is more consistent across diverse methods [14, 15].

TAD boundaries are typically enriched with actively expressed genes and active epigenetic states [7, 9, 13]. The disruption of TAD boundaries is commonly associated with ectopic gene interactions, gene dysregulation, and a spectrum of abnormal phenotypes including cancer and neurodevelopmental disorders [16–20]. The categorical investigations of boundaries have highlighted notable disparities among different boundary types. For instance, research by Gong *et al.* [21], characterized genome-wide boundaries based on insulating strength, revealing that boundaries with a high insulation score predominantly correlate with CTCF binding and super-enhancers. Similarly, studies by McArthur *et al.* [22], focusing on the stability of boundaries across cell types, have demonstrated that stable boundaries are further enriched with complex-trait heritability, evolutionary constraints, and are intricately linked with diseases. A recent study [23] providing a multi-species comparative analysis of TAD boundaries has underscored their evolutionary conservation, suggesting the strategic role of ultra-conserved boundaries in evading rearrangements associated with breakages of synteny. Hence, the contribution of TAD boundaries to biological functions warrants an exhaustive and detailed exploration. Notably, the 3D structure of the genome exhibits a close association with DNA damage repair mechanisms [24–26], with boundary regions being particularly susceptible to breaks and subsequent repair [27]. The coexistence between active transcriptional regulation and vulnerability to damage is a critical piece in the puzzle of genomic stability and regulation, the exact mechanisms of which remain unclear.

Our research focuses on the phenomenon where multiple nested TAD structures share a single boundary. The shared utilization of boundaries may play a critical role in the preservation of hierarchical structure and the regulation of gene activity. Specifically, we investigate whether these shared boundaries represent distinct genomic features or functionalities and whether these features are indicative of meaningful biological disparities. Following the description by An *et al.* [13], we employ a stratified approach to define high-level boundaries as those shared by multiple TADs based on the maximum number of TADs on either side. This stratified characterization aims to broaden our understanding of transcriptional regulation and DNA damage repair within the context of the 3D genomic landscape.

**Figure 1:**
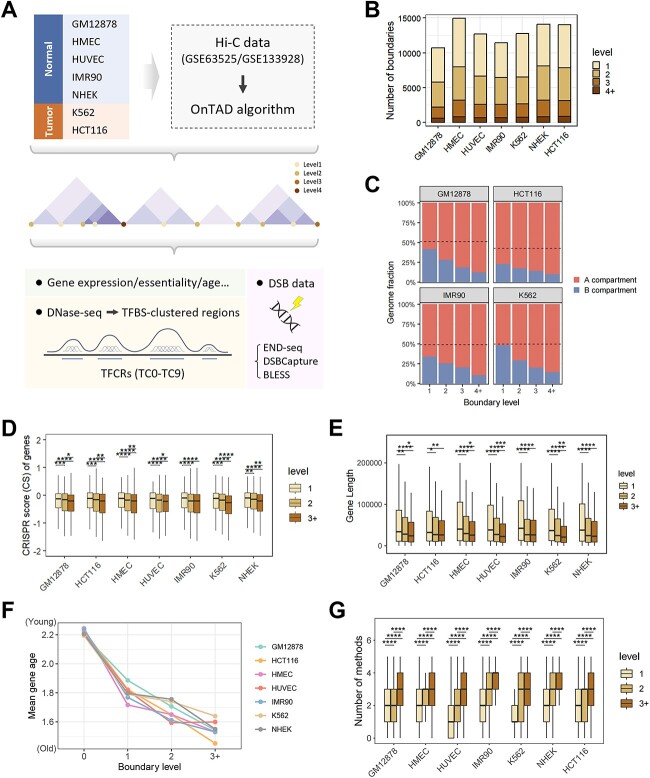
Definition of the TAD boundary levels and its correlation with gene characteristics. (A) Flowchart of the study is shown, along with an illustration of the TAD boundary levels. The boundary levels are defined as the maximum number of TADs that use a boundary on either its left or right side. (B) Statistics of the levels of TAD boundaries in seven cell lines. (C) Barplot showing the fraction of the genome (y axis) assigned to different level boundaries (x axis). The black dashed line represents the genome-wide level control. (D) Distribution of CRISPR score values for genes whose promoter (+/− 1 kb from TSS) overlap with TAD boundaries. Genes are classified by the level of TAD boundaries. (E) Distribution of gene length. Genes are classified by the level of TAD boundaries. ^*^P < 0.05, ^*^^*^P < 0.01, ^*^^*^^*^P < 0.001, ^*^^*^^*^^*^P < 0.0001, using Wilcoxon-test. (F) Correlation between boundary levels and gene age, with high-level genes appearing to be older on average. Level 0 refers to genes that are not located at the boundary. In each cell line, ANOVA test *p* value < 2.2e-16. (G) Boxplot showing the number of different level boundaries recognizable by other methods in individual cell lines.

Through the integration of Hi-C data from seven cell lines, various epigenetic data, and reliable DSB data, we conducted a systematic exploration of the complex regulatory patterns at high-level boundaries (Fig. 1A). Employing the optimized OnTAD methodology [13], we delineated the hierarchical structure of TADs and elucidated the stratified narrative of TAD boundaries. Our detailed analysis reveals that high-level boundaries are not mere demarcations within the genomic landscape but serve as active centers, enriched with functionally pivotal genes and high-complexity TFBS-clustered regions (TFCRs). Meanwhile, high-level boundaries demonstrate a pronounced enrichment of DNA double-strand breaks (DSBs), as evidenced by the overlapping of DSB peaks and DSB motif analysis. These ‘hub-boundaries’, characterized by their high level, intricate transcription factor-binding, and conservation, are identified as focal points strongly associated with the DNA repair process, heralding a paradigm of ‘sharing the danger, and sharing the fix’ within the transcriptional regulation landscape. In summation, our comprehensive exploration through the genomic intricacies reveals that the shared stewardship of TAD boundaries offers a profound lens through which to view the functional landscape of the genome. It enriches our understanding of the interplay between damage repair mechanisms and the complex 3D structure of the genome, presenting not only a narrative of complexity but also of harmony and resilience in the face of genomic perturbations.

## Results

### Stratification of TAD boundaries illuminates gene characteristics

To embark on a meticulous exploration to delineate TADs and their boundaries, we collected high-quality genome-wide chromatin conformation (Hi-C) data from Rao *et al.* [4] and Johnstone *et al* [28], spanning both normal and tumor cell lines (Fig. 1A). Our methodology involved the deployment of the OnTAD algorithm [13], processing Hi-C contact matrices at a fine-grained 10 kb resolution to derive nested TAD structures. To further investigate the significance of boundaries frequently shared by multiple TADs, we stratified TAD boundaries according to the maximum frequency used by adjacent TADs on either left or right side (Fig. 1A, middle panel), as described by An *et al.* [13]. The nested TAD structure alters the use of boundaries, with boundaries classified as level 1–4. Notably, boundaries shared by four or more TADs were categorized into level 4. We counted the number of boundaries assigned to each category for each of the seven cell lines (Fig. 1B). To further assess the resolution invariance of TAD boundaries, we performed additional analyses at multiple resolutions, from 5 to 50 kb. We present a detailed statistical comparison of TAD boundary levels across these resolutions in seven cell lines (Supplementary Fig. 1A), highlighting the distribution and consistency of boundaries at each resolution. Notably, high-level TAD boundaries identified at 10 kb resolution were found to be largely preserved at the 5 kb resolution (Supplementary Fig. 1B), but less so at 50 kb resolution (except for the HCT116 cell line, which is still ~50%), which may be directly influenced by the low number of level3+ boundaries identified at 50 kb resolution. This tiered boundary landscape laid the groundwork for our subsequent analysis.

We evaluated the genetic and epigenetic attributes of these stratified boundaries. Comparing the annotated compartment map to the identified TAD boundaries, we discerned that boundaries were prone to reside within the active A compartments, with increased percentage of higher-level boundaries assigned to A compartment intervals (Fig. 1C). Expectedly, this localization to A compartments denotes the active state of the boundary shared by more TADs. To assess the relationship between genetic characterization and boundary sharing, we classified genes according to the overlap of their promoters (+/− 1 kb from TSS) with different level boundaries.

We observed, consistent with preceding studies [7, 13], gene expression levels soared at boundary sites, escalating further with increasing boundary levels (Supplementary Figs. 2A, B, C). We then focused on gene essentiality, characterized by CRISPR scores (CS) measured in previous studies by Wang *et al.* [29]. In short, a lower score indicates higher gene essentiality, and vice versa. Our analysis revealed that, in line with the trend in gene expression levels, genes at boundaries have higher essentiality, and the essentiality increases with higher boundary levels (Fig. 1D, Supplementary Figs. 2D, E). Additionally, previous research has reported that gene length affects the stability of genetic switch dynamics, thereby influencing gene expression dynamics [30]. Therefore, we examined the length differences of genes at different boundaries. We noticed that genes at higher-level boundaries are shorter (Fig. 1E). Furthermore, the age attribute of genes to some extent reflects their inter-species evolutionary conservation [31, 32]. Based on the characteristic defined in previous studies [31], we explored its relationship with boundary levels. Specifically, we partitioned protein-coding genes into age classes according to their phyletic origin (age) of genes, defined by the evolutionarily most distant species group where homologs can be found. The smaller age values represent the earlier the gene origin, indicating the older genes. We confirmed that genes at higher-level boundaries are older, indicating earlier origins and higher conservation (Fig. 1F). However, when comparing based on whether genes are located at boundaries, we found that genes at boundaries are generally longer and have more transcript variants (Supplementary Figs. 2F, G). This was also expected, ensuring their more complex transcriptional functionality compared to non-boundary genes. However, when stratifying boundaries, the trend becomes more differentiated, highlighting the importance of detailed exploration through boundary stratification.

**Figure 2:**
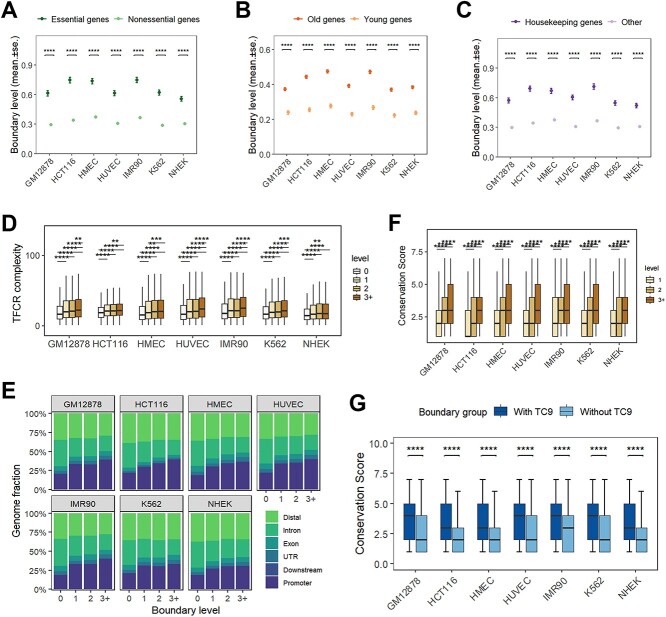
High-level TAD boundaries are enriched for functional genes, TFBS-clustered regions, and conservation across cell lines.(A-C) Comparison of boundary levels of different genes (A, essential genes; B, old genes; C, housekeeping genes). Gene boundary levels are based on the boundary overlapped by the gene promoter (+/− 1 kb from TSS). (D) Boxplots showing the complexity of TFCRs (TFBS-clustered regions) overlapping with different levels of TAD boundaries. (E) The distribution of genomic annotations for TFCRs classified by the level of TAD boundaries. (F) Correlation between boundary level and conservative score. Higher level boundaries show a higher degree of conservation across cell lines. The definition of conservation score is detailed in Supplementary Fig. 3E. (G) Categorizing the boundaries by the presence or absence of TC9 revealed higher conservation scores for the former. ^*^^*^P < 0.01, ^*^^*^^*^P < 0.001, ^*^^*^^*^^*^P < 0.0001, using Wilcoxon-test.

**Figure 3:**
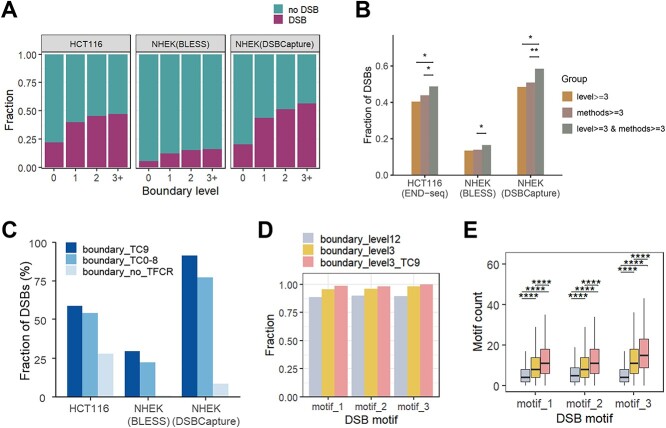
Enrichment of DSB events at high-level boundaries, especially those with high-complexity TFBS-clustered regions.(A) Barplots showing the fraction of different level boundaries overlapping the DSB peaks. Level0 refers to random genomes, i.e. bins with the same number of boundaries randomly sampled from the genome of the respective cell line. (B) Enrichment of DSBs at different grouped boundaries. ^*^P < 0.05, ^*^^*^P < 0.01, using Fisher’s exact test. (C) Enrichment of DSBs at boundaries with/without TFBS-clustered regions. The boundaries with high-complexity TFBS-clustered regions (TC9) show significantly higher overlap with the DSB peaks. (D) Enrichment of DSB motifs (top3) at different categories of boundaries in the HCT116 cell line. *De novo* motif mining for the DSB peaks and the comparison of motif enrichment were performed using MEME suite. (E) High-level boundaries, especially those overlapping with high-complexity TFBS-clustered regions, are enriched for DSB motifs. ^*^^*^^*^^*^P < 0.0001, using Wilcoxon-test.

To ensure the reliability of our TAD boundary identification, we utilized five distinct reference TAD calling methods: Insulation score [11], ArrowHead [4], TopDom [33], deDoc [34], and HiTAD [35]. We then analyzed the recognition of TAD boundaries across these varied methods. Our findings indicate that a significant proportion of boundaries are consistently identified by multiple methods, with higher-level boundaries demonstrating even greater consensus (Fig. 1G, Supplementary Figs. 3A, B, Supplementary Table 2). Furthermore, we conducted the benchmarking analysis of TAD boundaries recognized by a majority of reference methods, highlighting the enrichment of these boundaries for key performance metrics (Supplementary Fig. 4). Specifically, the group of boundaries identified as high-level and also recognized by a majority of methods (level > =3 & methods > = 3) exhibited significant CTCF enrichment, elevated gene expression, and increased gene essentiality, compared to the group identified by methods > = 3 alone. This enrichment provides further validation for our TAD boundary analysis and strengthens the characterization of level > =3 boundaries. These additional analyses substantiate the robustness of our TAD boundary identification and contribute to a deeper understanding of their biological significance.

In summary, our preliminary exploration verified that the stratification of boundaries correlates with numerous gene characteristics. Genes at boundaries shared by more TADs are more actively expressed, have higher essentiality, and are shorter and more conserved. These gene characteristics may have significant implications for the stability of cellular functions at boundary locations. These results also reflect that our stratification strategy for boundaries can to some extent reflect biological differences and allow for valuable analysis.

### High-level boundaries: a crucible of complex regulation

Our inquiry extended into the realm of functional elements congregating at high-level boundaries. Our earlier work [36] finely categorized coding genes by ascending CS values (CS0-CS9), where CS0 was composed of essential genes, representing genes that are actively expressed and play vital roles in important biological processes. As expected, we observed the enrichment of essential genes at high-level boundaries (Supplementary Fig. 5A). Comparing based on the level of boundaries overlapped by gene promoters, we found essential genes are at higher level than non-essential genes (Fig. 2A, Supplementary Fig. 5D). We performed the same analysis for old genes (age of 1, refer to genes with earlier phyletic origin and evolutionary conservation [31]) and housekeeping genes [37], with results indicating a higher congregation of these functionally pivotal genes at high-level boundaries (Figs. 5B, C, Supplementary Figs. 5B, C, E), hinting at a center of transcriptional activity and complexity.

**Figure 4:**
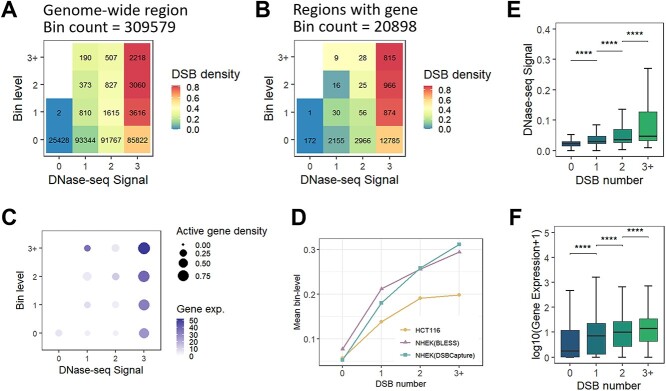
Genome-wide DSB enrichment validate correlation with structural and epigenetic features. (A) Heatmap shows comparison of DSB density among genomic bins classified by two categorical variables (x-axis: ascending DNase-seq signal; y-axis: bin level according to the maximum number used as a boundary by TADs). Text annotations indicates the number of bins in the subgroup, and color represents the mean value of DSB density (i.e. the number of DSBs in each bin) in the subgroup. Genome-wide analysis was performed using the same resolution as calling TAD, 10 kb. (B) Comparison of DSB density among bins with gene overlapping. (C) Scatterplot of DNase-seq signal and bin level. Circle size indicates the number of active genes (FPKM >5), and color represents the average gene expression in the subgroup. (D) Comparison of mean level of genome bin grouped by number of DSBs. In each cell line, ANOVA test *p* value < 2.2e-16. (E–F) Boxplots comparing the characteristics (E: DNase-seq signal; F: local gene expression) of bins categorized by number of DSBs. ^*^^*^^*^^*^P < 0.0001, using Wilcoxon-test.

**Figure 5:**
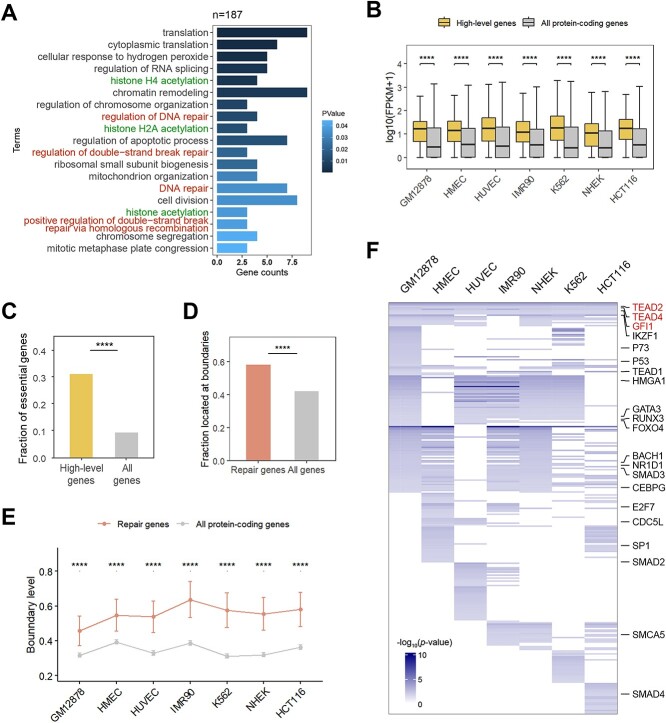
High-level and cell line-stabilized boundaries seem to exceptionally involve in repair events. (A) GO enrichment analysis showing the GO terms (*p*-value <0.05) associated with genes conserved at high-level boundaries (level2+) in seven cell lines. (B) Expression levels of the genes conserved at high-level boundaries in seven cell lines (high-level genes, n = 187) were significantly higher compared to all protein-coding genes (n = 20,242). ^*^^*^^*^^*^P < 0.0001, using Wilcoxon-test. (C) Barplot showing enrichment of essential genes in high-level gene list. ^*^^*^^*^^*^P < 0.0001, using Fisher’s exact test. (D) Barplot indicated repair-related genes (from Hussmann *et al.*, n = 476) were significantly enriched at boundaries relative to all protein-coding genes. The plot showed the fraction of genes whose promoter (+/− 1 kb from TSS) overlap with TAD boundaries in the gene lists. ^*^^*^^*^^*^P < 0.0001, using Fisher’s exact test. (E) Repair-related genes (n = 476) were on average located at the boundaries of higher level in all seven cell lines. ^*^^*^^*^^*^P < 0.0001, using Wilcoxon-test. (F) Motif matches enriched at the high-level (level3+) with high-complexity TFBS-clustered region (TC9) boundaries in each cell line. The repair-related motif has been shown by text labels. Motifs enriched in all seven cell lines are labeled red. The motif enrichment *p*-value was derived from Tomtom tool in MEME suite with default setting.

Differences in non-coding sequences might alter the binding of transcription factors on the genome, thereby affecting transcriptional regulation [38–40]. Previous research has found that transcription factor binding sites (TFBSs) are highly clustered in biological genomes [41–43], possibly reflecting synergistic regulation processes of gene expression. In our effort to unravel the intricacies of transcriptional regulation, we delved into the distribution of TFBSs-clustered regions (TFCRs). Using the computational method developed in our previous work [44] to identify TFCRs through Gaussian kernel density estimation, we identified TFCRs of varying complexity based on DNase-seq data collected from various cell lines. With ascending TFBS complexity, the number of identified TFCRs located within promoters increased (Supplementary Fig. 6A). TFCRs, when categorized by the level of TAD boundaries they are located at, showed a positive correlation between level and complexity (Fig. 2D). Additionally, as the level increased, the proportion of TFCRs located within promoters also increased (Fig. 2E). Based on complexity, each TFCR set was divided into 10 equally-sized categories (TC0-TC9). We confirmed the enrichment of high-complexity TFCRs at higher-level boundaries (Supplementary Figs. 6B, C, D). Our findings illustrate increasing TFCR complexity at higher-level boundaries, suggesting a more elaborate regulatory mechanisms in these genomic sectors.

Previous research indicates TAD boundaries that are stable across different cell types are subject to evolutionary constraints and are enriched for heritability [22]. Therefore, we examined whether our stratification of boundaries indicates differences in boundaries present across different cell lines. We visualized the boundary collections of seven cell lines (Supplementary Fig. 6E) and assigned a conservation score to each boundary based on its appearance in the collections. The results showed that boundaries of high levels have higher conservation scores, indicating a more stable presence across different cell lines (Fig. 2F, Supplementary Fig. 6F). Additionally, boundaries co-localizing with TC9 (the highest complexity TFCR) also showed significantly higher conservation across cell lines (Fig. 2G).

In conclusion, our results suggest that boundaries shared by more TADs, i.e. high-level boundaries, mediate complex, and more active transcriptional regulation. Furthermore, our analysis illuminated the evolutionary resilience of these regions, with higher-level boundaries exhibiting a remarkable conservation across diverse cell lines, evidence of their fundamental role in genomic integrity.

### Breaks are enriched at high-level boundaries

The interplay between TAD boundaries and DNA DSBs emerged as a focal point of our investigation. TAD boundaries are instrumental in the spatial clustering and isolation of damaged regions [25, 45, 46], thereby facilitating effective repair mechanisms. Additionally, functional genes closely associated with TAD boundaries are susceptible to DNA DSBs (Supplementary Figs. 7A, B, C). Our stratification for boundaries aims to delve into the correlation between different TAD boundaries and the occurrence of DSBs in the context of chromatin 3D structure. Utilizing publicly available DSB sequencing data [47, 48], we analyzed the proportion of DSB peaks overlapping at distinct level boundaries. Relative to non-boundary regions (refer to level 0), the enrichment of DSBs at TAD boundaries is evident, with moderately increasing at the boundaries shared by more TADs (Fig. 3A). For each category, we calculated the average number of overlapping DSB peaks and depicted the DSB levels surrounding the boundaries, yielding robust results (Supplementary Figs. 7D, E, F). We extended our analysis to compare the enrichment of DSBs within TAD boundaries of varying levels of recognition among reference TAD callers. As detailed in the Methods, we categorized the TAD boundaries based on whether they were identified as level > =3 and/or recognized by a majority of reference methods (methods > = 3). Our analysis revealed that TAD boundaries falling into the category of level > =3 & methods > = 3 demonstrated a heightened enrichment of DSB loci (Fig. 3B) This suggests that the consensus among multiple methodologies confers an additional layer of reliability upon our findings, indicating that these boundaries are not only structurally significant but also functionally relevant in the context of genomic stability. The enrichment of breaks at boundaries aligns with prior research [24, 49], while the detailed stratification of boundaries indicates certain variations in DSB enrichment.

The aforementioned analyses suggest that higher-level boundaries are linked to more complex transcriptional regulation, prone to harbor higher complexity TFCRs. Thus, we further probed whether boundaries co-localizing with TFCRs exhibit the variation in the concentration of damage. As hypothesized, boundaries with TFCRs, especially those with high-complexity TFCRs, demonstrate an enrichment of DSBs (Fig. 3C, Supplementary Fig. 7G), in line with the notion that genomic regions actively engaged in transcriptional regulation may be more vulnerable to damage.

We further differentiated the boundaries into low-level boundaries, high-level boundaries, and high-level boundaries co-localizing with TC9 for comparison. Due to the sparsity of DSB peaks relative to the whole-genome regions, we utilized the MEME suite [50] to identify DSB-associated motifs and reported the matching of these motifs in different boundary sequences. Analyses indicate that high-level boundaries with TC9 show a stronger association with DSB motifs, which maintained the validity across various datasets (Figs. 3D, E, Supplementary Fig. 7H).

These findings illuminate the differential susceptibility of boundaries to DSBs. High-level boundary regions, particularly those enriched with TFCRs and active regulation, tend to be more fragile. Understanding these nuances enhances the comprehension of the relationships between genomic structure and function, providing insights into the mechanisms of genome stability and potential vulnerabilities within the chromatin architecture.

### Genome-wide DSB enrichment validate correlation with structural and epigenetic features

To comprehensively understand the preferential occurrence of DNA DSBs events across the genome and validate their correlation with structural and epigenetic features, we partitioned the entire genome at the resolution of TAD calling (10 kb). Two categorical variables were determined: bin levels based on the frequency of TADs used as boundaries and DNase-seq signals within bins (see Methods). DNase-seq demonstrated reliability and strength in identifying active regulatory elements on the genome [51, 52]. There is a significant overlap between higher-level TAD boundaries and DHSs (Supplementary Fig. 8A), suggesting a shared regulatory mechanism. We compared DSB density (number of DSB peaks overlapping each bin) and the average level of DSB signals per bin in different categories of bins in the HCT116 cell line (Fig. 4A, Supplementary Fig. 8B). We observed a positive correlation between DSB occurrence and both levels indicated by the x and y-axes, with bins in the top-right category exhibiting the highest propensity to damage. The finding held true when comparing only genomic regions with genes, showing even more significant differences (Fig. 4B). This conclusion remained robust when analyzing DSB peaks called using alternative sequencing methods in the NHEK cell line (Supplementary Figs. 8C, D). Subsequently, we examined the expression levels of genes located within genomic bins. We discovered that genes in regions characterized by the highest degree of accessibility and serving as boundaries for more TADs exhibit the highest expression levels, which also most enriched for active genes (FPKM >5) (Fig. 4C). These findings align with our analyses in the preceding text, indicating that genome-wide breakages are not random but exhibit preferential patterns.

Furthermore, we conducted comparisons based on the number of DSBs in genomic regions. Due to the discrepancy in DSB-captured methods, the number of DSB peaks varies substantially, even within the same cell line (Supplementary Fig. 9A). Firstly, we confirmed the correlation between bin-level and the number of DSBs within bins. Analyses of three DSB datasets consistently showed an increase in bin-level on average with an increasing number of DSBs (Fig. 4D). Additionally, regarding the epigenetic features of the genome, genome accessibility as well as the expression level of relevant genes are higher in DSB-prone regions (Figs. 4E, F, Supplementary Figs. 9B, C). These results ensure the robustness of our analysis in reflecting the general characteristics of preferential regions for breakage occurrence across different cell types.

Our comprehensive genome-wide analysis of DSB occurrence revealed a compelling correlation with both structural and epigenetic features, notably TAD boundary sharing as well as regulatory sequence variations indirectly reflected by chromatin accessibility. This association underscores the non-random nature of genomic breakages, highlighting preferential patterns that align with regions of heightened transcriptional and regulatory activity.

### Hub-boundaries show exceptional enrichment of DNA repair-mediated genes

We have elucidated that the boundaries shared by multiple TADs signify active transcription and are more conserved across cell types, as well as being more susceptible to damage. However, it remains unclear as to how cells respond to damage to ensure their normal functionality. To address this query, we performed Gene Ontology analysis on genes located at high-level (level 2+) boundaries in all seven cell lines, categorized as high-level genes (n = 187, Supplementary Data 1). The results showed that these boundary genes, besides exhibiting functions related to chromatin organization, chromatin remodeling, and chromosome segregation, also displayed significant enrichment for DNA repair-related functions (Fig. 5A, Supplementary Data 2). Furthermore, the enrichment analysis suggested the gene set was associated with histone acetylation, which is closely related to DNA damage repair, affecting chromatin accessibility and structure, thereby regulating the cellular response to DNA damage [53–56]. Notably, we observed that genes conserved at the high-level boundary include DNA topoisomerase II beta-binding protein 1 (*TOPBP1*), which is widely known to play a key role in maintaining genome stability and regulating DDR-associated signaling pathways [57, 58], and *BRCA2*, which is involved in DNA repair and is essential for maintaining genome integrity and resisting DNA damage [59, 60]. We then extended the boundaries by one bin to each side, and genes robustly located at high-level boundaries (n = 827, Supplementary Data 3) also exhibit associations with DNA repair, though to a slightly lesser degree (Supplementary Fig. 10A, Supplementary Data 4). These findings may point out high-level boundaries, despite being high prevalence spots for DSBs, efficiently coordinate repair-related transcription to resist the impact of damage, ensuring proper gene regulation. Relative to all protein-coding genes, the expression levels of these high-level genes are significantly higher, with a greater proportion being essential genes (Figs. 5B, C).

Additionally, we noted a set of genes (n = 476) involved in DNA repair or related processes from published research [61]. In comparison to all protein-coding genes, repair-related genes are significantly enriched at boundaries (Fig. 5D, Supplementary Fig. 10B). In all seven cell lines, repair-related genes were, on average, located at higher level boundaries (Fig. 5E). This further underscores the co-localization of high-level boundaries with repair-related genes. The observed patterns in our results might highlight an intricate evolutionary adaptation within the chromatin structure, suggesting a delicate balance. Higher-level TAD boundaries, while more susceptible to breaks, are strategically enriched with repair-related genes. This configuration enables more efficient repair over extensive areas, illustrating an advanced evolutionary strategy that maintains genomic stability by coupling vulnerability with enhanced repair capabilities.

To directly characterize the sequence features of the classified boundary regions, we determined the motif enrichment at boundaries shared repeatedly with the presence of TC9 in several cell lines (Fig. 5F). Our analysis identified 277 motifs recognized in at least one of the cell lines (Supplementary Data 5), including numerous factors related to DNA damage repair (labeled in text in Fig. 5F), notably the TEAD family proteins and GFI1 factor, which present at boundaries across all seven cell lines. TEAD has been reported to co-localize with DNA damage-induced nuclear foci and play a role in the DNA damage repair process [62, 63]. GFI1 is a transcriptional regulator expressed in lymphoid cells, mediating the post-translational modification of proteins involved in DNA repair [64]. Furthermore, we explored the enrichment of motifs with varying width and found that these boundary regions were stably enriched for multiple repair-related motifs even when the parameters were changed (Supplementary Fig. 10C, D), reinforcing the robustness of our results that high-level boundaries with a high-complexity TFCRs are involved in DNA repair and genome stability.

This evokes the notion of the ‘Transcription Factory’ [65], a bustling hub where multiple stimulus-responsive genes and a complex array of transcription factors are pulled together for efficient and synergistic transcription. Similarly, we term boundaries frequented by multiple TADs, inclined to mediate complex regulation, as ‘hub-boundaries’. In contrast to the definition in An *et al.* [13], our definition of hub-boundaries is more restrictive, assuming not only bustling centers of transcription but also pivotal in DNA repair processes.

### Sharing the danger, and sharing the fix

**Figure 6:**
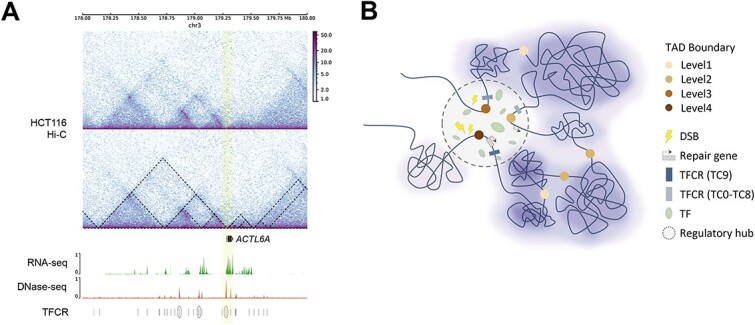
Hub-boundaries are vulnerable to breaks, but also prone to repair to ensure positive regulation. (A) Representative example of ‘hub-boundary’ and multi-omics landscape. The captured region is chr3, 178.0–180.0 Mb near the *ACTL6A* gene. The upper panels show nested TAD structures. The high-level boundary (marked by yellow stripe), i.e. shared by multiple TADs, is accompanied with high gene expression, high chromatin accessibility and high-complexity TFBS-clustered regions (marked by elliptical circles). (B) Schematic diagram showing the complex regulatory process at hub-boundaries. The dark brown circle indicates a high-level boundary, termed fragile sites undergo DSBs, while multiple transcription factors are recruited to coordinate the repair process, maintaining proper gene regulation patterns.

In the aforementioned analysis, we illustrated that hub-boundaries exhibit a propensity for breakage, yet possess an inherent capability for swift repair, ensuring positive regulation. We specifically focused on repair-related genes, exemplified by *ACTL6A*, a gene proven to promote the repair of cisplatin-induced DNA damage in tumor therapy [66]. We scrutinized the TAD hierarchy as well as gene expression abundance and epigenetic features within the 2/3 Mb genomic region vicinity surrounding the *ACTL6A* gene via the Genome Browser (Fig. 6A, Supplementary Fig. 11A). Our observations revealed that *ACTL6A* resides at boundaries shared by multiple TADs, along with high gene expression, high chromatin accessibility, and high-complexity TFCRs (marked by elliptical circles).

This exemplifies a typical scenario of a gene situated at the ‘hub-boundary’, signifying intricate regulatory processes linked to DNA repair. We delineated the regulatory landscape at hub-boundaries (Fig. 6B), where dark brown circles signify high-level boundaries—essentially ‘fragile sites’ susceptible to DSBs. We propose that, for enhanced efficiency, a ‘transcription factory’-like model operates at high-level boundaries. Due to their high accessibility, these boundaries may share a central regulatory hub, where functional proteins are further concentrated, facilitating active transcription of genes implicated in DNA repair, counteracting the damage caused by the enriched DSBs at these sites and thus preserving genomic stability. The model emphasizes the cellular strategy to execute a sophisticated response to maintain genome integrity amidst DNA damage within the 3D chromatin context.

## Discussion

The advent of advanced chromatin conformation capture technologies has markedly deepened our understanding of the hierarchical structure of mammalian chromatin, uncovering the nuanced interplay within TAD and sub-TAD structures. Our research probes into the realm of shared TAD boundaries, critical for upholding the hierarchical chromatin structure and modulating gene activity. By integrating high-resolution Hi-C data, chromatin accessibility insights, and DNA DSBs data across diverse cell lines, we have dissected the regulatory landscape at high-level TAD boundaries.

Our comprehensive analysis of TAD boundaries and their correlation with gene characteristics and structural integrity of the genome offers novel insights into the intricate architecture of chromatin organization and its functional implications. The stratification of TAD boundaries, as revealed in our study, does not merely demarcate genomic regions but intricately influences gene expression, essentiality, and evolutionary conservation. The predilection of higher-level boundaries to align with active A compartments underscores a nuanced interplay between spatial genome organization and transcriptional activity. The observed enrichment of gene expression and essentiality at these junctures highlights the pivotal role of boundary stratification in modulating gene function. The evolutionary vestiges, marked by the presence of older genes at higher-level boundaries, further attest to the conserved nature of these genomic regions, suggesting their fundamental role in maintaining genomic stability across diverse cell lines.

The exploration into the high-level boundaries, designated as hub-boundaries, unravels a complex regulatory scaffold where transcription factors and stimulus-responsive genes converge. The TFCRs at these boundaries illuminates a sophisticated regulatory network, potentially orchestrating gene expression dynamics. The enrichment of essential and housekeeping genes at these loci signifies a concentrated hub of biological activity, pivotal for maintaining cellular homeostasis.

Intriguingly, the stratification of TAD boundaries bears a profound connection with the genomic landscape integrity. The enriched occurrence of DSBs at high-level boundaries underscores a paradoxical nature—regions that are critical for transcriptional regulation and gene expression are also the hotspots for genomic instability. This observation prompts a reflection on the evolutionary trade-offs between the need for dynamic gene regulation and the imperative of maintaining genomic integrity. The revelation that hub-boundaries are not only susceptible to damage but also inherently equipped for efficient DNA repair mechanisms offers a glimpse into the cellular resilience against genomic perturbations. The presence of DNA repair-related genes and motifs at these boundaries suggests a preemptive adaptation, possibly to counterbalance the inherent fragility of these regions.

Our study, thus, presents a multifaceted portrayal of TAD boundaries, depicting them as dynamic interfaces where gene regulation, evolutionary conservation, and structural integrity intersect. The notion of the ‘Transcription Factory’, as evoked by our findings, aptly encapsulates the orchestrated synergy of transcriptional regulation, DNA repair, and genome stability mechanisms at these ‘hub’ locations.

There are also certain limitations in our study that warrant further exploration in subsequent research. First, the absence of experimental validation underscores the necessity for empirical studies to substantiate our computational findings and theoretical predictions. Exploring a wider array of tissue types would also enhance the generalizability of our results, providing a more comprehensive understanding of chromatin dynamics across different biological contexts. Additionally, delving into single-cell level analysis could unveil the nuances of chromatin organization and repair mechanisms that bulk analyses might overlook, revealing cell-to-cell variability and its implications for gene regulation and genomic stability.

Despite these limitations, our work lays the groundwork for a deeper understanding of the chromatin functional landscape. It emphasizes the complexity of biological processes occurring at TAD boundaries and highlights their role as critical nodes in the maintenance of genomic stability and the execution of cellular functions. The exploration provides a valuable perspective on how cells integrate various regulatory layers to ensure the fidelity of genetic information and the robustness of DNA repair responses. More profoundly, this study offers a scaffold for unraveling the complexities of genomic diseases, where disruptions in the structural and functional integrity of TAD boundaries could manifest in aberrant gene expression patterns and compromised cellular functions. Our study paves the way for future investigations into the therapeutic potential of modulating boundary characteristics, offering a promising frontier in precision medicine and genome engineering.

## Materials and methods

### Data source

High-throughput Chromosome Conformation Capture (Hi-C) data were obtained from Rao *et al.* [4] and Johnstone *et al.* [28]. DNaseI hypersensitive sites sequencing (DNase-seq) data were obtained from Duke and UW ENCODE groups (details in Supplementary Table 1). DNA DSBs data were collected from GEO database (GSE129529 and GSE78172). The processed RNA sequencing (RNA-seq) data were downloaded from the ENCODE consortium [67] (https://www.encodeproject.org/). Gene annotations were obtained from the GENCODE data (V15).

### Identification of hierarchical TADs

The paired-end reads were processed using HiC-Pro (v3.1.0) [68] (https://github.com/nservant/HiC-Pro) and the contact matrices were generated at resolutions of 10 kb and normalized using the iterative correction and eigenvector decomposition (ICE) [69]. We obtained hierarchical TADs using OnTAD software from normalized Hi-C matrix data. In brief, OnTAD first uses an adaptive local minimum search algorithm to identify candidate TAD boundaries. Then, OnTAD assembles TADs from the candidate boundaries using a recursive algorithm. We take the 10 kb resolution Hi-C interactions matrix as input and use sparseToDense scripts to convert the sparse matrix into a dense matrix, and then calculate the hierarchical TAD structure with the parameters: - penalty 0.1 -minsz 3 -maxsz 200.

### TAD boundary level determination

The methods for calculating the TAD boundary level in our study were the same as those reported previously [13]. In detail, if a boundary was shared by no more than one TAD on each side, the boundary was classified as level 1; if a boundary was shared by no more than two TADs on each side, the boundary was classified as level 2; by analogy, if there were no more than four TADs on each side of a boundary, then it could be upgraded to level 4+. For instance, a boundary should be classified as level 4 if it was shared by three TADs to its left and four TADs to its right.

### Recognition of TAD boundaries across reference methods

Except for OnTAD, we run other five reference TAD calling approaches, including Insulation score (https://github.com/dekkerlab/crane-nature-2015), ArrowHead (https://github.com/aidenlab/juicer/wiki/Arrowhead), Topdom (https://github.com/HenrikBengtsson/TopDom), deDoc (https://github.com/yinxc/structural-information-minimisation), and HiTAD (https://pypi.python.org/pypi/TADLib). Considering a degree of inconsistency in the realm of TAD and sub-TAD identification, while the delineation of TAD boundaries is more consistent across diverse methods, we directly calculated the fraction of the current boundaries that overlap with the boundaries identified by reference methods. To facilitate a clear and comparative analysis, we categorized the TAD boundaries into three groups based on their level and the number of identifying methods: level > =3, methods > = 3, and level > =3 & methods > = 3 for various performance metrics comparison. Due to the high precision of the 10 kb resolution, we appropriately expanded the boundaries by one bin each on the left and right when performing the overlap analysis, i.e. a single boundary spanning a 30 kb genomic region.

### Gene enrichment analysis

To evaluate the activity of gene expression, we downloaded the RNA-seq data from ENCODE. Genes with FPKM >5 were deemed as active genes. The list of essential genes was taken from the categorization study by Chen *et al.* [36]. Housekeeping genes (n = 2176) are from Hounkpe *et al.* [37]. Genes with promoters (defined as the upstream and downstream 2 kb region of the transcription start site) overlapping the boundary are classified by the level of the boundary. The genome assembly version was hg19.

### Identification of TFCRs

First, the TFBSs were identified by FIMO [70]. The position-specific weight matrices of transcription factors were downloaded from CIS-BP databases [71]. The genomic sequences under the open chromatin regions from DNase-seq data were used as inputs for FIMO with a custom library of all motifs to scan for motif instances at a *p*-value threshold of 10^−5^. Then, an established method [44] was used to identify TFCRs by performing the Gaussian kernel density estimations across the genome (with a bandwidth of 300 bp centered on each TFBS). Each peak in density profile was considered a TFCR. To determine the complexity of each TFCR, the Gaussian kernelized distances from each peak that contributed at least 0.1 to its strength were determined. The complexity of each TFCR was determined by the quantity and proximity of the contributing TFBS. We combined motif instances based on the TF family information from CIS-BP to calculate the complexity of TFCRs. The window for each TFCR was determined by finding the maximum distance (in bp) from the TFCR to a contributing TF and then adding 150 bp (one-half of the bandwidth). Each window was centered on the TFCR. The identified TFCR was grouped into 10 groups (TC0-TC9) based on their complexity from low to high. The ChIPseeker package [72] was used to annotate the relative position of TFCRs.

### Motif analysis

Using MEME suites [50] (https://meme-suite.org/meme/tools/meme) to search motif, DSB sites binding motif analysis was performed. We use the DSB signal enrichment peak BED files as input. The number of target motifs was set to 3. The same parameters were used for all transcription factors to facilitate comparability of data. Use FIMO (https://meme-suite.org/meme/tools/fimo) to identify boundary sequences matching the DSB motifs. The motif enrichment of the boundary is also realized by the MEME suite. We scanned DNA binding motifs to identify sequence features for high-level boundary co-located with TC9 in each cell line, setting the parameters as follows: -nostatus -mod zoops -nmotifs 5 -minw 6 -maxw 30 -objfun classic -revcomp -markov_order 0. Use Tomtom (https://meme-suite.org/meme/tools/tomtom) to implement comparative analysis of motifs. The tool can compare one or more motifs against a database of known motifs (HOCOMOCO-v11 [73]), with the parameter of -verbosity 1 -min-overlap 5 -dist Pearson -evalue.

### DSB signal enrichment analysis

The profiles of stratified boundaries were identified using the center of the boundaries as anchors with binning the DSB signals in 5 kb bins ±50 kb up and downstream of the anchor. Results were plotted by deepTools2 [74].

### Genome-wide DSB enrichment

To comprehensively understand the preferential occurrence of DSBs across the whole-genome and validate their correlation with structural and epigenetic features, we partitioned the entire genome at the resolution of TAD calling (10 kb). We partitioned genomic regions by two categorical variables, DNase-seq signal and bin level according to the maximum number of TADs used as boundaries. Specifically, we incrementally homogenized the DNase-seq signals into three categories and additionally grouped the no-signal regions into category 0. For each bin, we compute DSB densities as the number of overlapping DSB peaks. We additionally categorized genome bin by the number of overlapping DSBs to compare differences in distinct genomic features.

### A/B compartment calling

We used HiTC R package [75] to identify A/B compartments by performing principle component analysis on Hi-C contact map. The annotated compartment maps were compared to the identified TAD boundaries and the percentage of different levels of boundaries allocated to A/B compartment intervals was calculated.

### GO enrichment analysis

We performed GO enrichment analysis for genes conserved at high-level boundaries using DAVID [76] (https://david.ncifcrf.gov/), specific GO terms displayed in the Supplementary Data 2 and 4.

### Visualization

The browser snapshots, including Hi-C heatmap, TAD hierarchy diagram, and multiple epigenomic tracks were plotted with pyGenomeTracks [77].

**Key Points**

- The Role of Shared Topologically Associating Domain (TAD) Boundaries: The study underscores the critical role of shared TAD boundaries in maintaining the hierarchical structure of chromatin, essential for gene regulation and genomic integrity.
- Complex Regulatory Landscape Exploration: We integrated high-resolution Hi-C, chromatin accessibility, and DNA double-strand breaks data to reveal the intricate regulatory dynamics at high-level TAD boundaries, highlighting their architectural and functional significance.
- Genomic Stability and Therapeutic Potential: Findings reveal a balance between transcriptional activity and DNA repair at TAD boundaries, influencing genomic stability, with implications for understanding genomic diseases and developing targeted therapies.

## Supplementary Material

S1_bbae306

S2_bbae306

S3_bbae306

S4_bbae306

S5_bbae306

S6_bbae306

S7_bbae306

S8_bbae306

S9_bbae306

S10_bbae306

S11_bbae306

Supplementary_Tables_bbae306

Supplementary_Figures_legend_bbae306

Supplementary_data_bbae306

Additional_files_bbae306
