# Supplementary figures and images for: Stratifying TAD boundaries pinpoints focal genomic regions of regulation, damage, and repair
Source: Brief Bioinform. 2024 Jun 27;25(4):bbae306. doi: 10.1093/bib/bbae306 (PMC11210073; doi:10.1093/bib/bbae306)

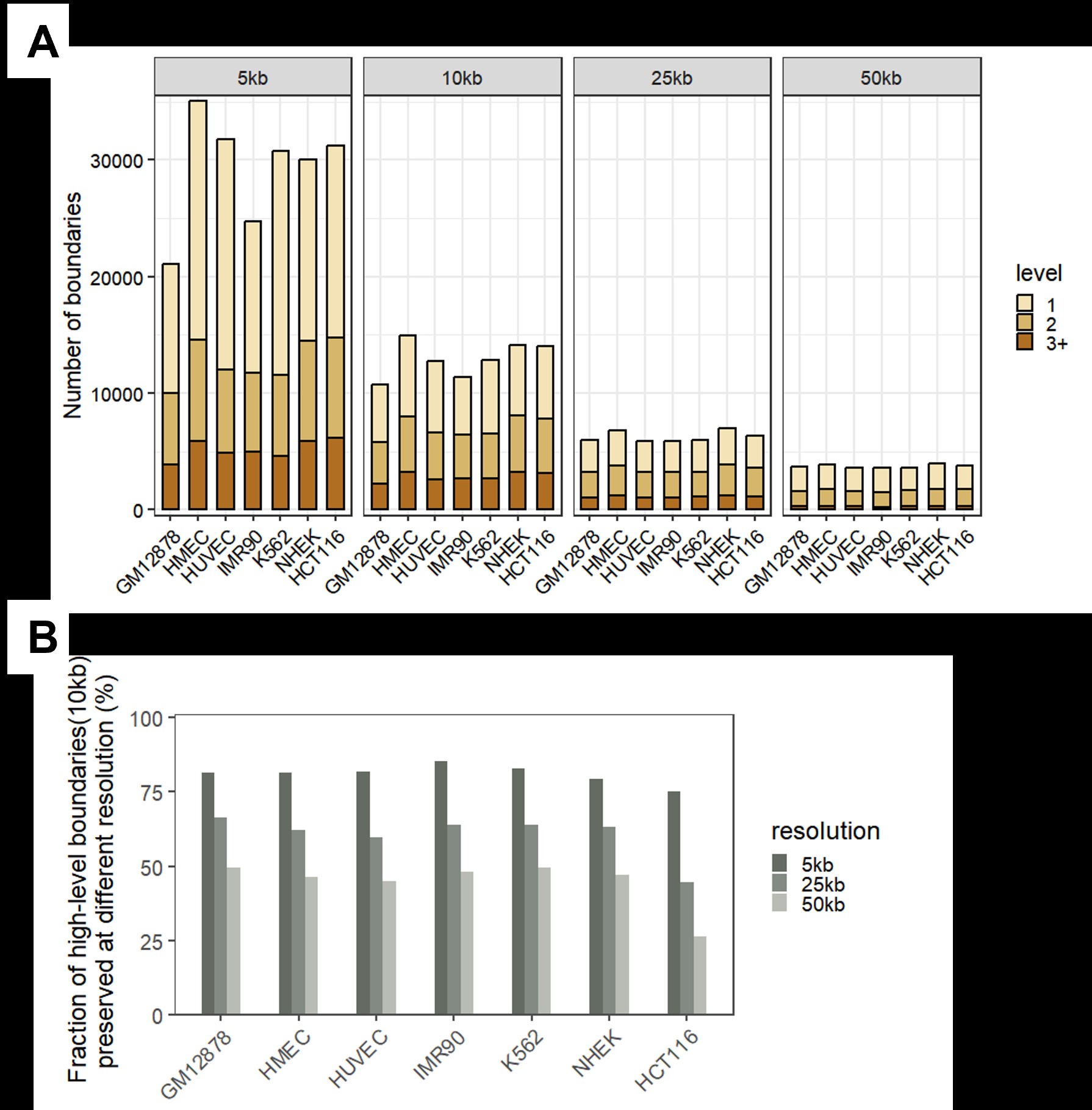

Supplement: S1_bbae306 [file s1_bbae306.jpeg]

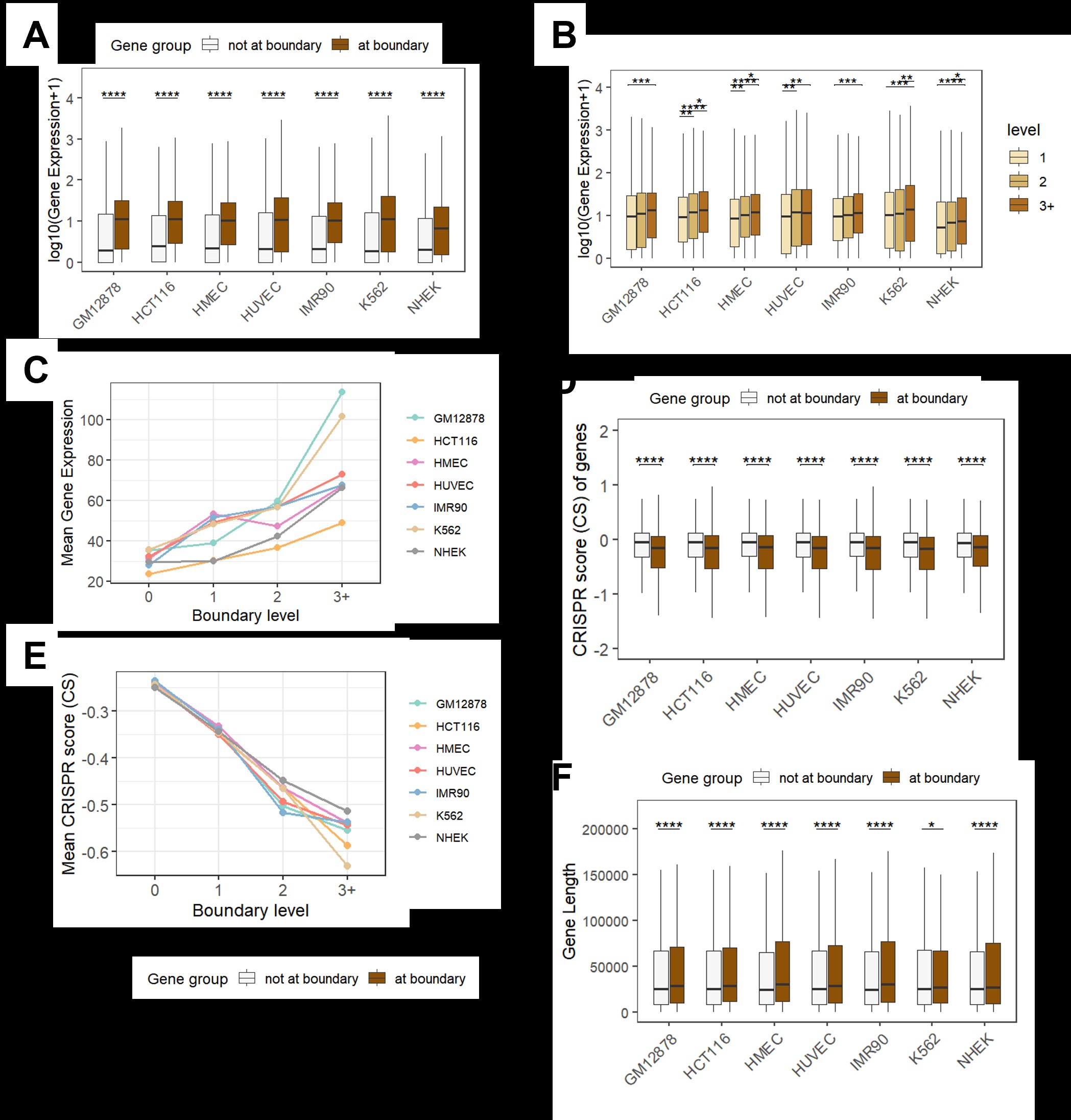

Supplement: S2_bbae306 [file s2_bbae306.jpeg]

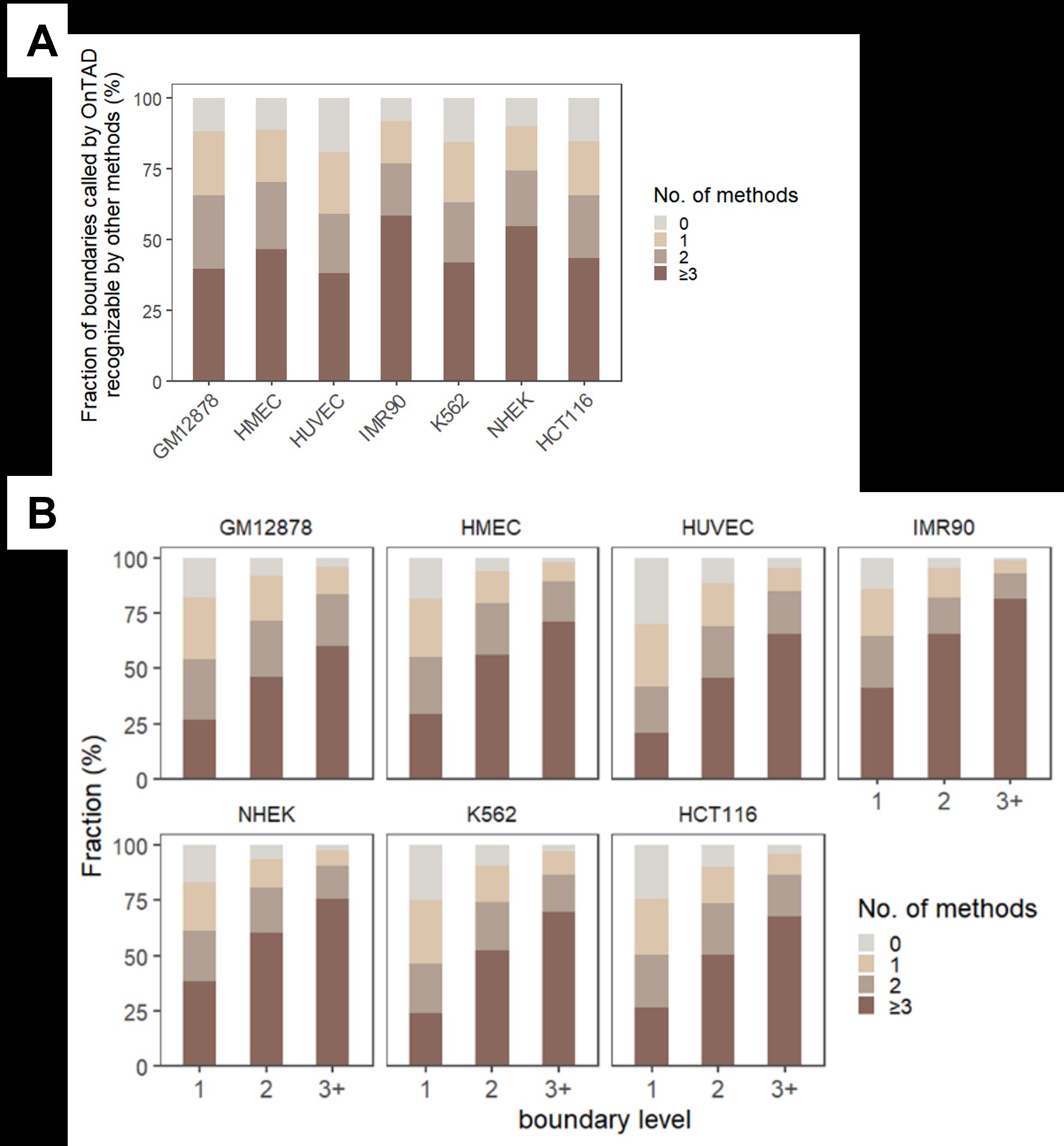

Supplement: S3_bbae306 [file s3_bbae306.jpeg]

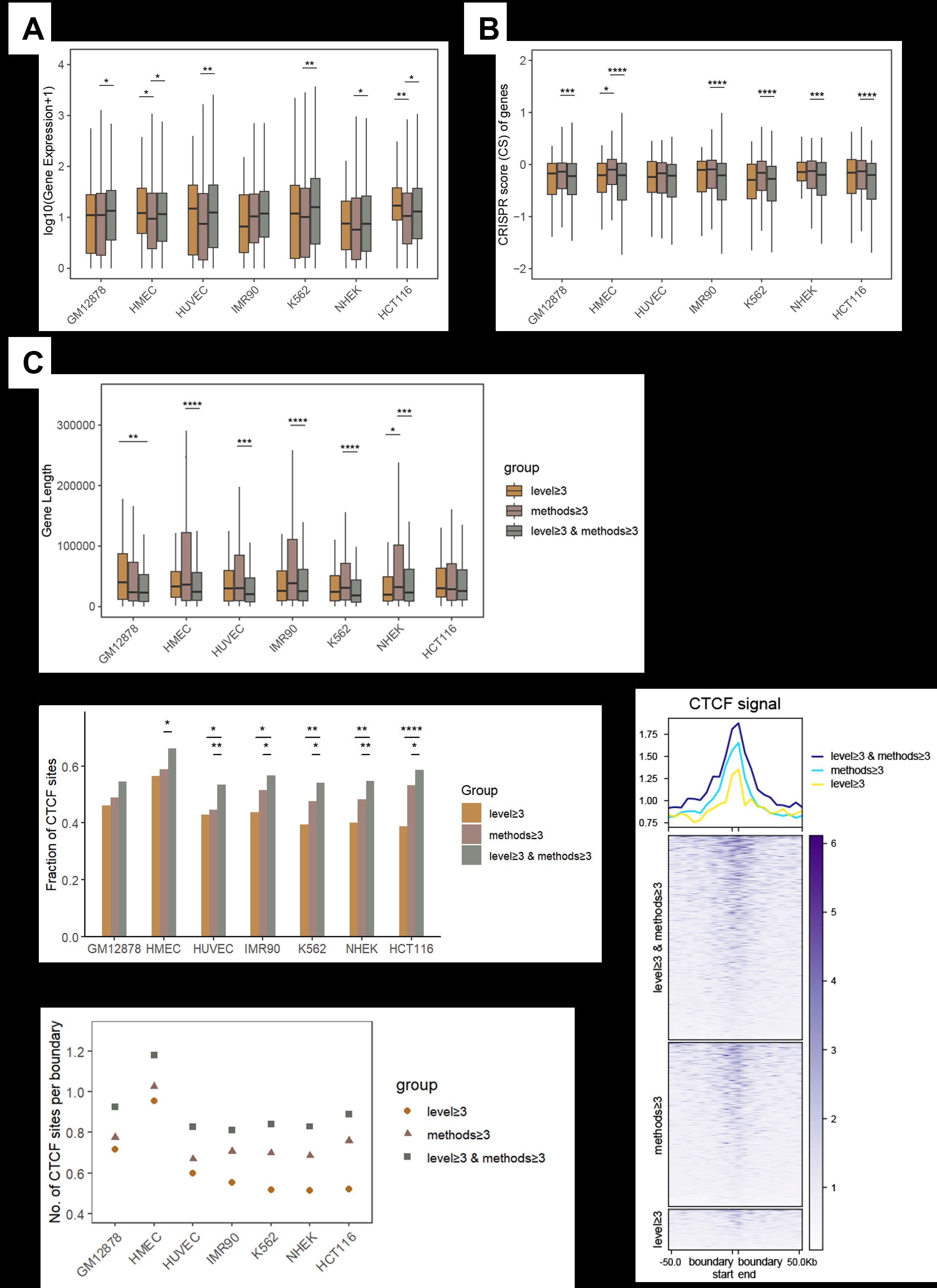

Supplement: S4_bbae306 [file s4_bbae306.jpeg]

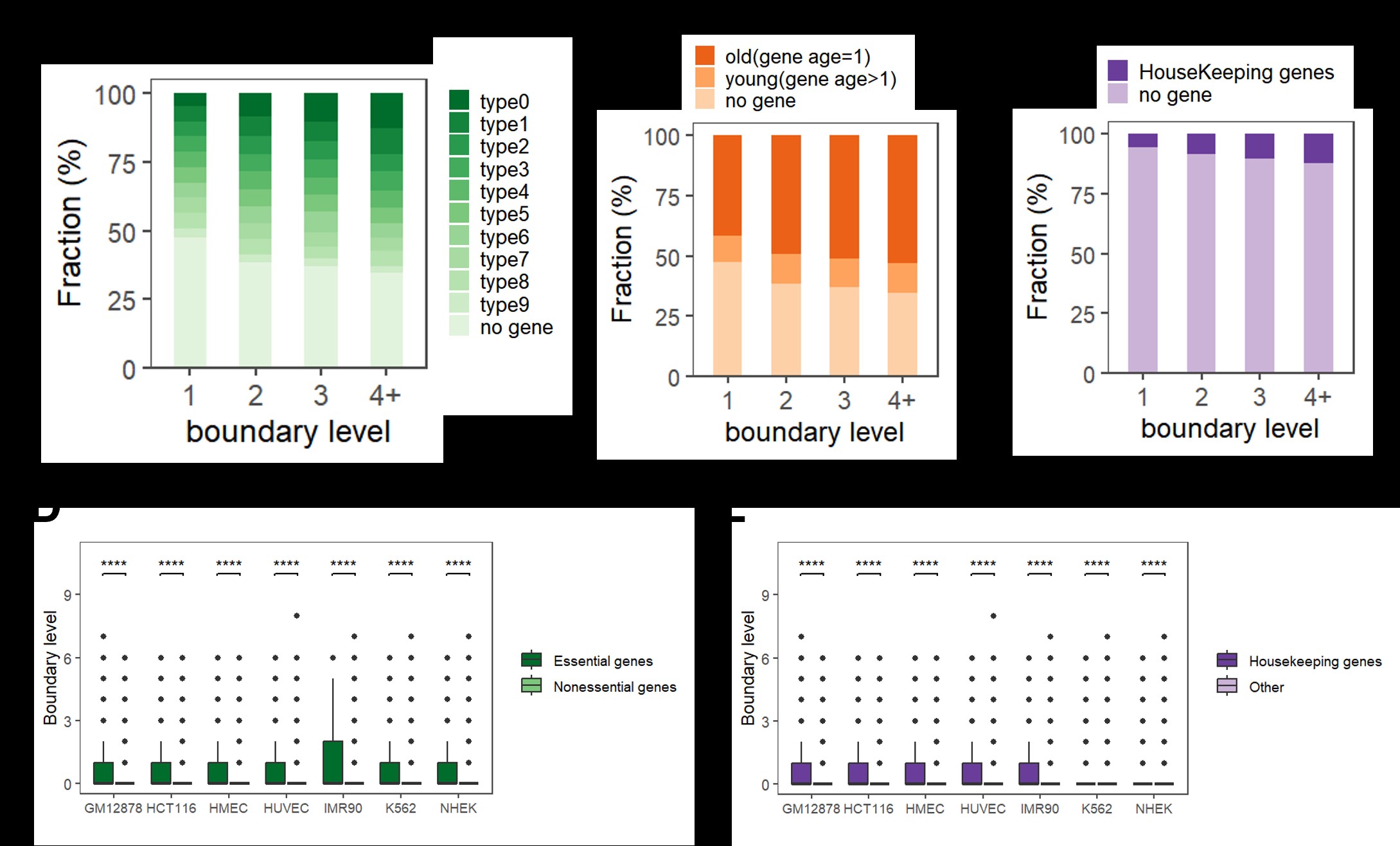

Supplement: S5_bbae306 [file s5_bbae306.jpeg]

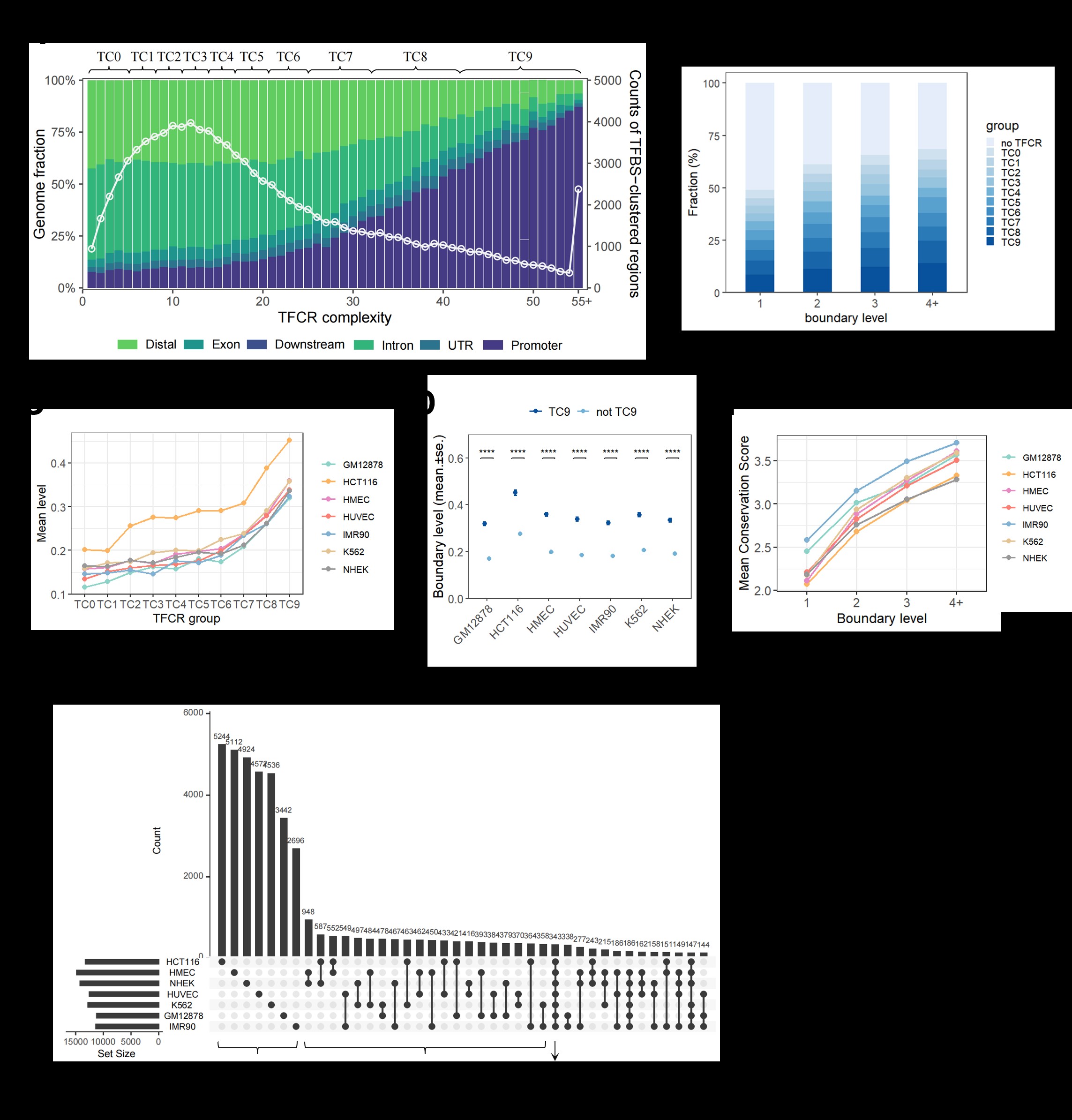

Supplement: S6_bbae306 [file s6_bbae306.jpeg]

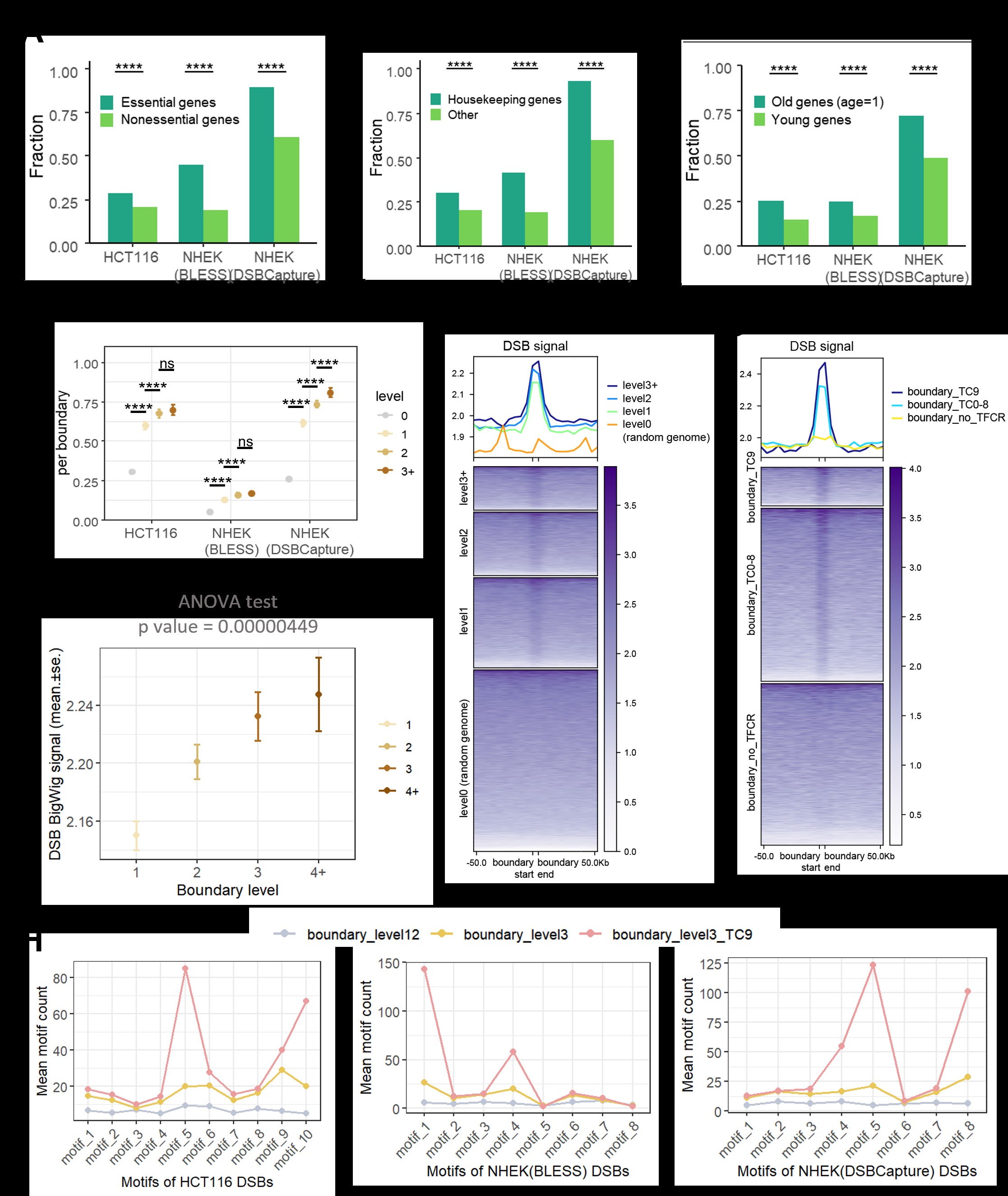

Supplement: S7_bbae306 [file s7_bbae306.jpeg]

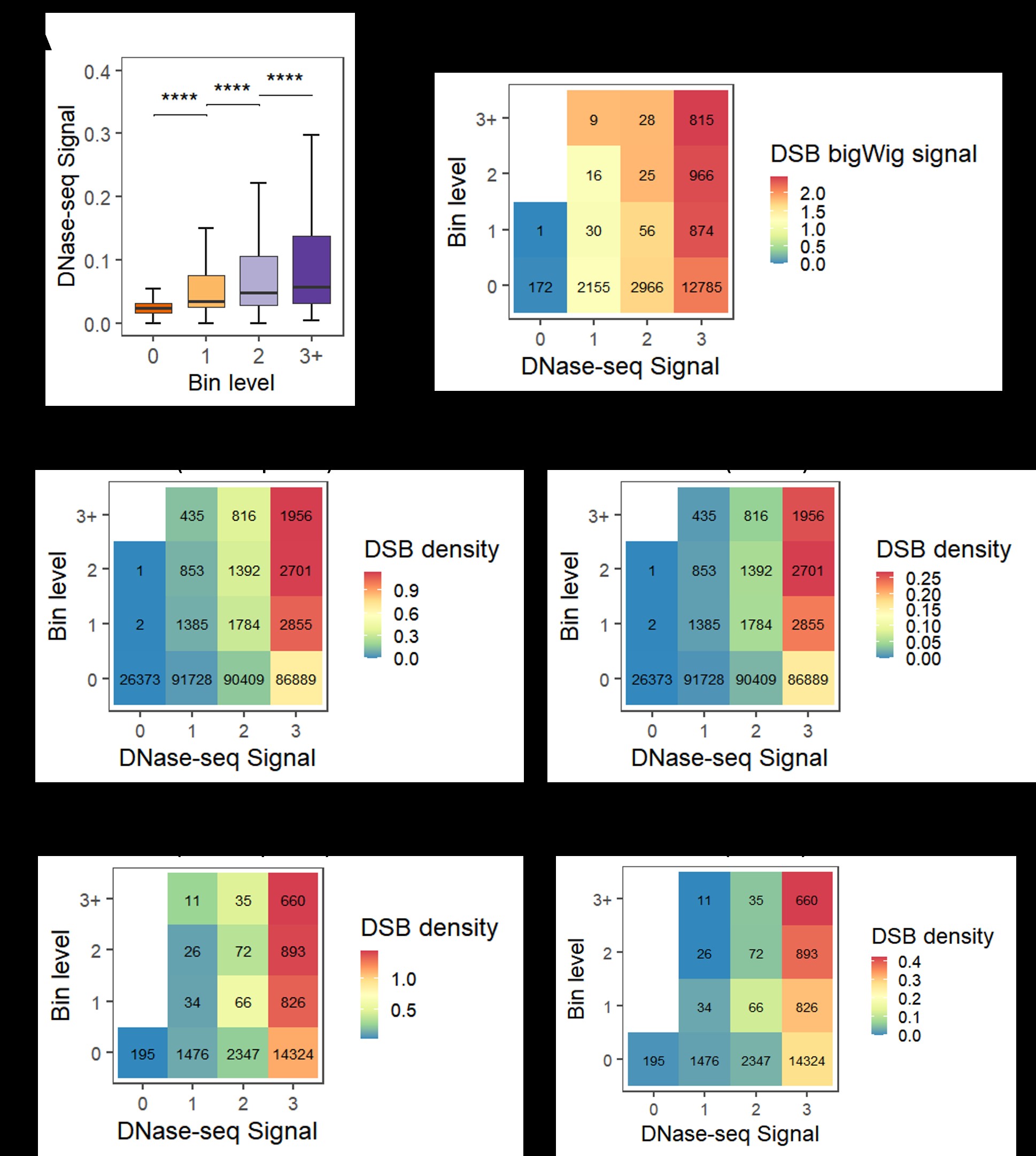

Supplement: S8_bbae306 [file s8_bbae306.jpeg]

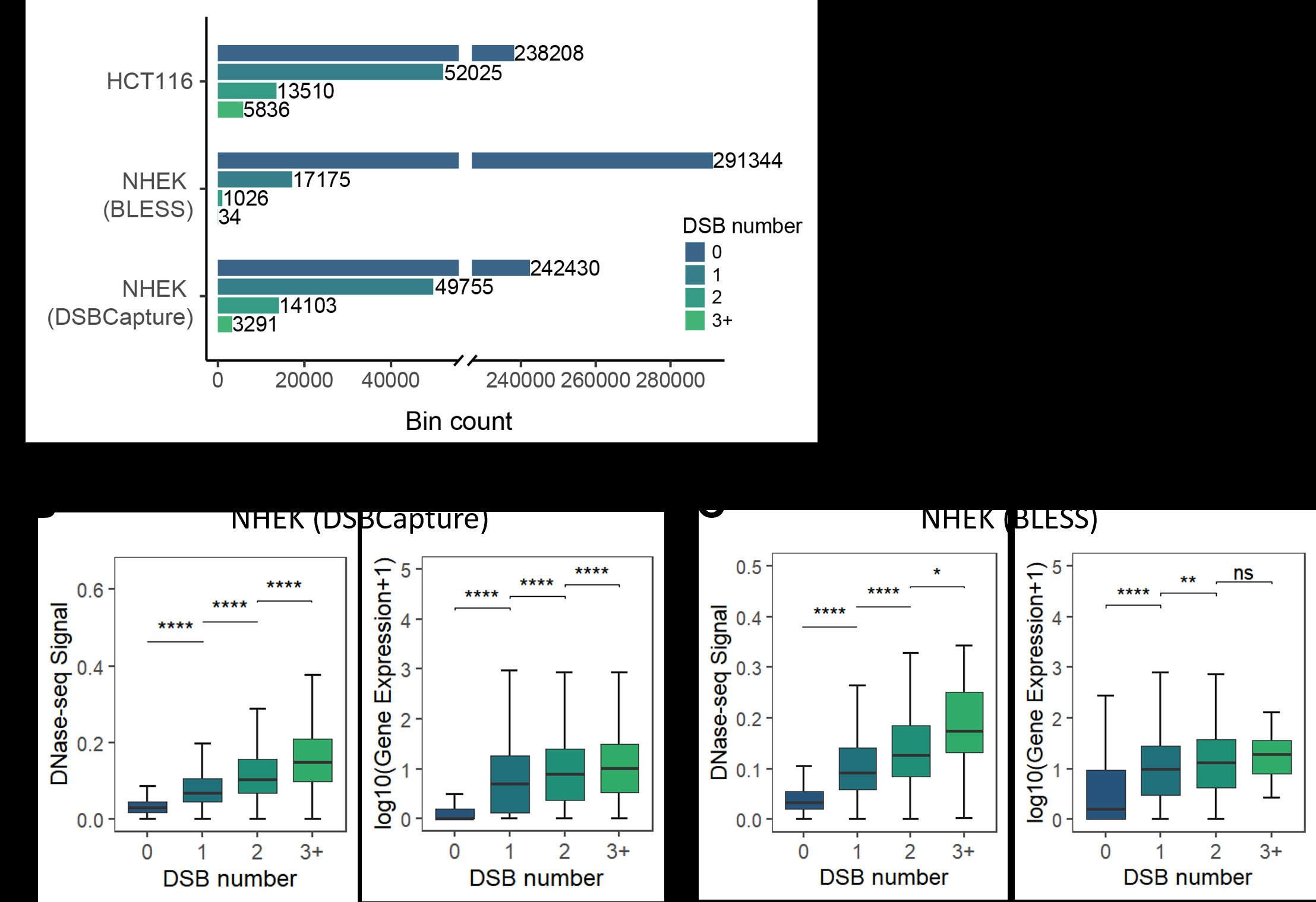

Supplement: S9_bbae306 [file s9_bbae306.jpeg]

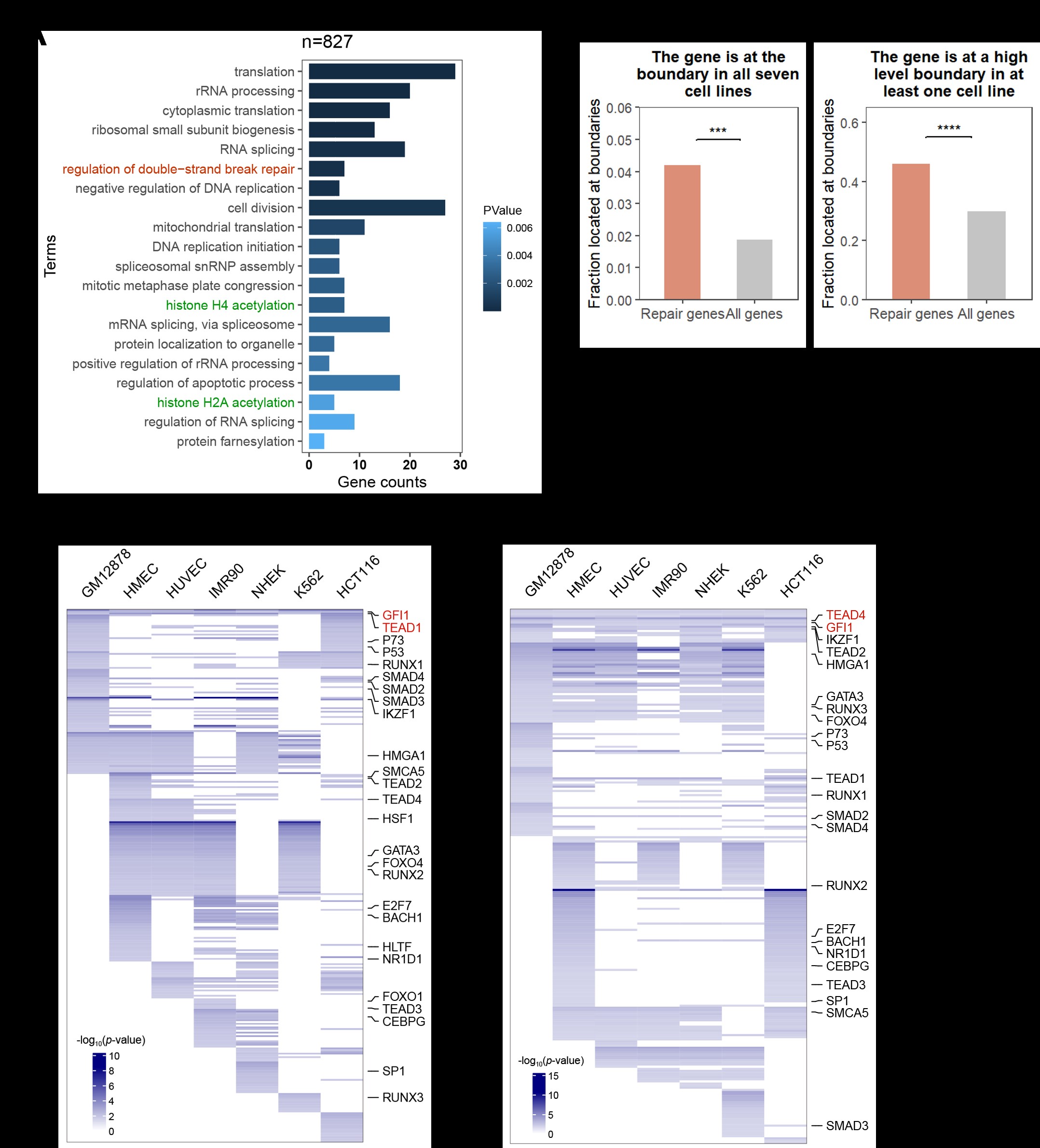

Supplement: S10_bbae306 [file s10_bbae306.jpeg]

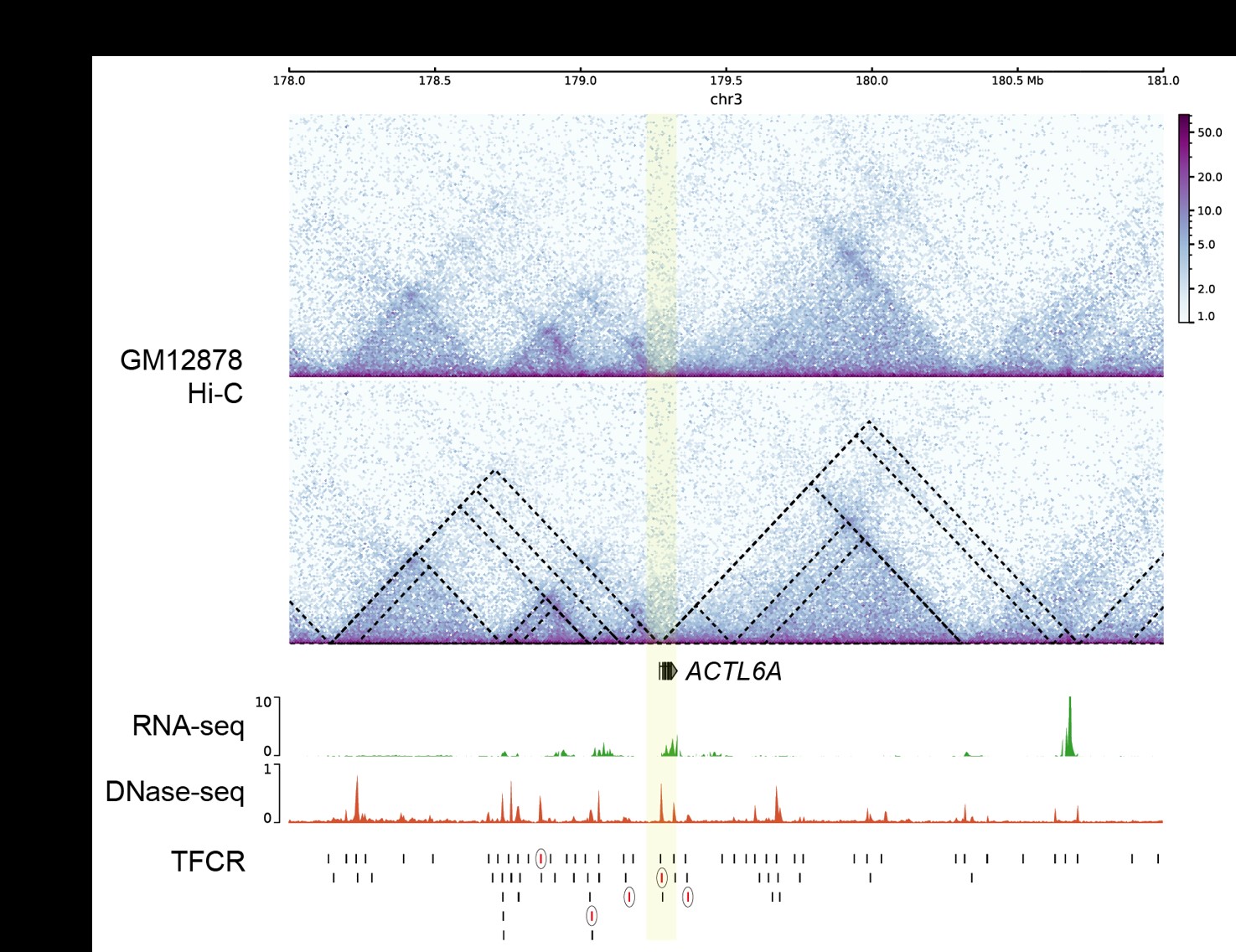

Supplement: S11_bbae306 [file s11_bbae306.jpeg]
