# Supplementary material for: Stratifying TAD boundaries pinpoints focal genomic regions of regulation, damage, and repair
Source: Brief Bioinform. 2024 Jun 27;25(4):bbae306. doi: 10.1093/bib/bbae306 (PMC11210073; doi:10.1093/bib/bbae306)
Supplement: Supplementary_Tables_bbae306 [file supplementary_tables_bbae306.pptx]

## Slide 1
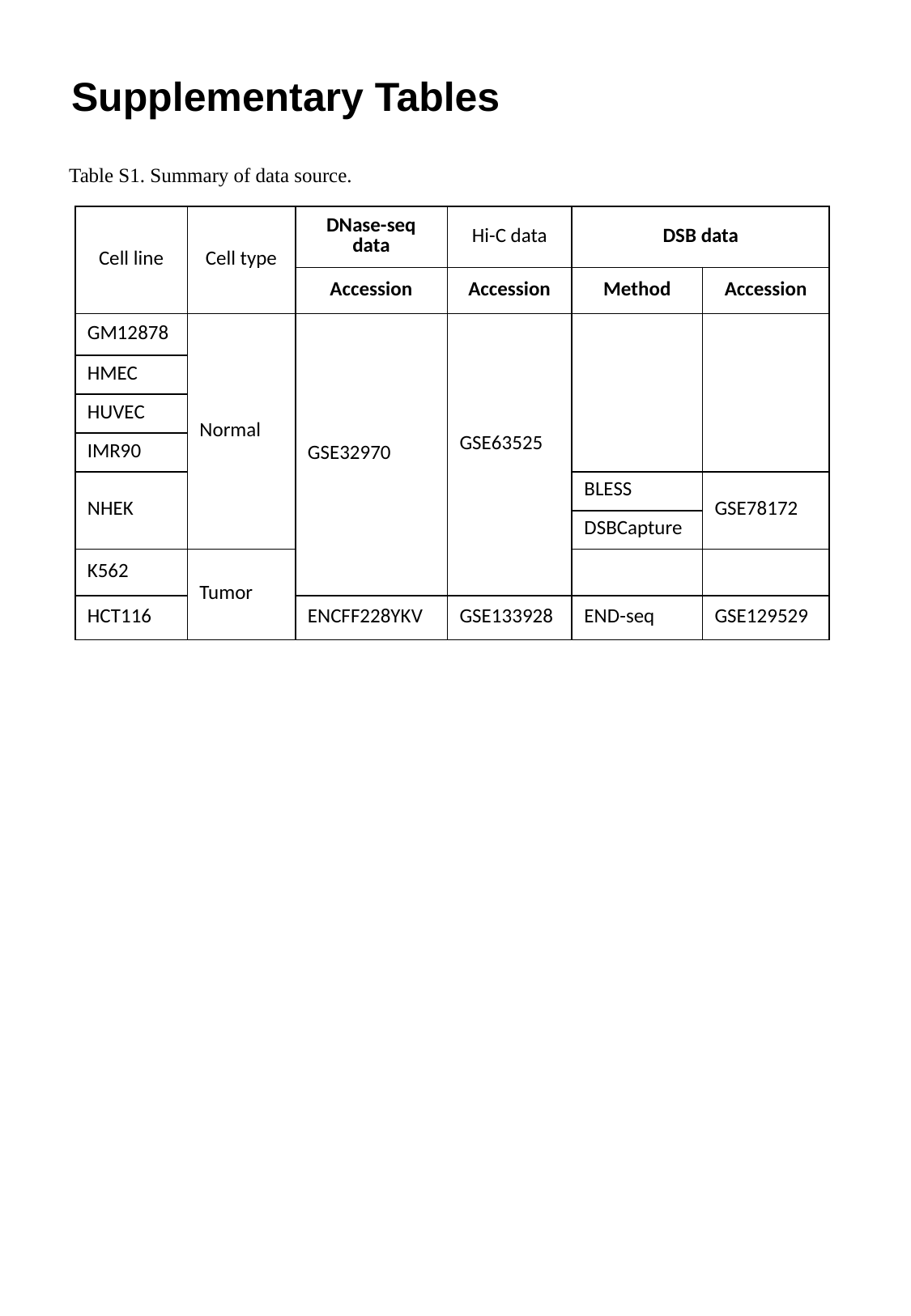

Supplementary Tables
Table S1. Summary of data source.
| Cell line | Cell type | DNase-seq data | Hi-C data | DSB data | |
| --- | --- | --- | --- | --- | --- |
| Cell line | | Accession | Accession | Method | Accession |
| GM12878 | Normal | GSE32970 | GSE63525 | | |
| HMEC | | | | | |
| HUVEC | | | | | |
| IMR90 | | | | | |
| NHEK | | | | BLESS | GSE78172 |
| | | | | DSBCapture | |
| K562 | Tumor | | | | |
| HCT116 | | ENCFF228YKV | GSE133928 | END-seq | GSE129529 |

## Slide 2
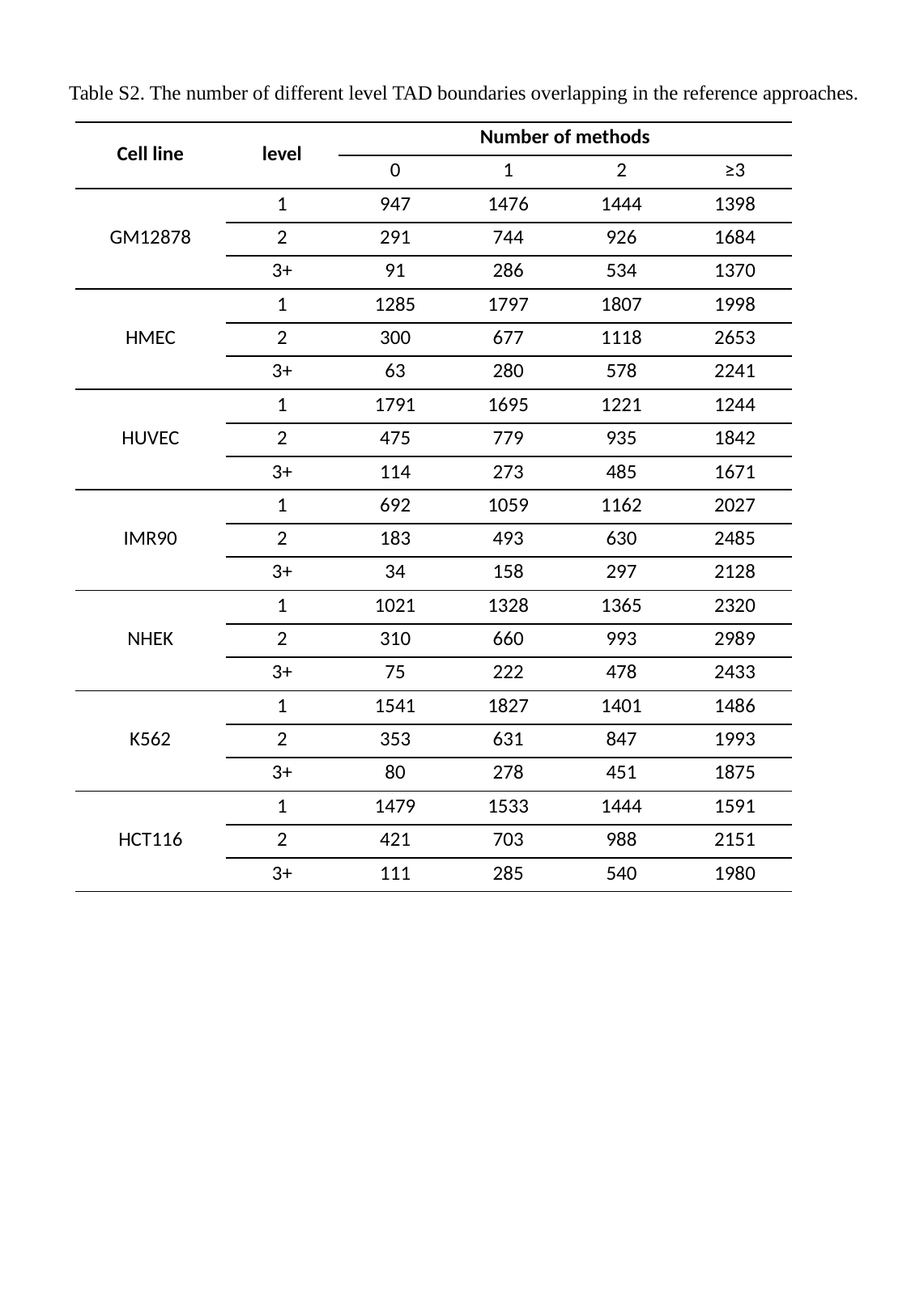

Table S2. The number of different level TAD boundaries overlapping in the reference approaches.
| Cell line | level | Number of methods | | | |
| --- | --- | --- | --- | --- | --- |
| | | 0 | 1 | 2 | ≥3 |
| GM12878 | 1 | 947 | 1476 | 1444 | 1398 |
| | 2 | 291 | 744 | 926 | 1684 |
| | 3+ | 91 | 286 | 534 | 1370 |
| HMEC | 1 | 1285 | 1797 | 1807 | 1998 |
| | 2 | 300 | 677 | 1118 | 2653 |
| | 3+ | 63 | 280 | 578 | 2241 |
| HUVEC | 1 | 1791 | 1695 | 1221 | 1244 |
| | 2 | 475 | 779 | 935 | 1842 |
| | 3+ | 114 | 273 | 485 | 1671 |
| IMR90 | 1 | 692 | 1059 | 1162 | 2027 |
| | 2 | 183 | 493 | 630 | 2485 |
| | 3+ | 34 | 158 | 297 | 2128 |
| NHEK | 1 | 1021 | 1328 | 1365 | 2320 |
| | 2 | 310 | 660 | 993 | 2989 |
| | 3+ | 75 | 222 | 478 | 2433 |
| K562 | 1 | 1541 | 1827 | 1401 | 1486 |
| | 2 | 353 | 631 | 847 | 1993 |
| | 3+ | 80 | 278 | 451 | 1875 |
| HCT116 | 1 | 1479 | 1533 | 1444 | 1591 |
| | 2 | 421 | 703 | 988 | 2151 |
| | 3+ | 111 | 285 | 540 | 1980 |

## Slide 3
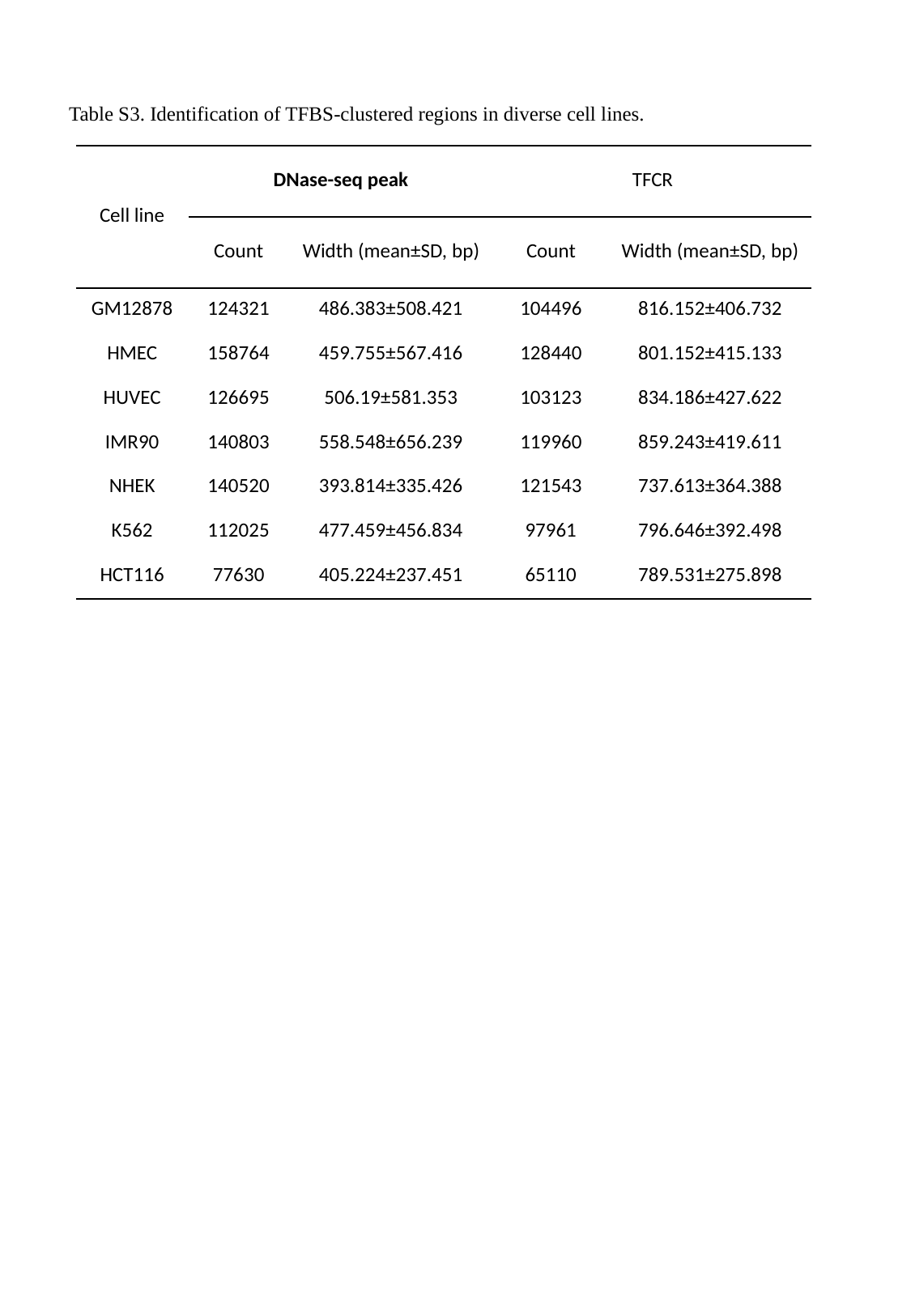

Table S3. Identification of TFBS-clustered regions in diverse cell lines.
| Cell line | DNase-seq peak | | TFCR | |
| --- | --- | --- | --- | --- |
| Cell line | Count | Width (mean±SD, bp) | Count | Width (mean±SD, bp) |
| GM12878 | 124321 | 486.383±508.421 | 104496 | 816.152±406.732 |
| HMEC | 158764 | 459.755±567.416 | 128440 | 801.152±415.133 |
| HUVEC | 126695 | 506.19±581.353 | 103123 | 834.186±427.622 |
| IMR90 | 140803 | 558.548±656.239 | 119960 | 859.243±419.611 |
| NHEK | 140520 | 393.814±335.426 | 121543 | 737.613±364.388 |
| K562 | 112025 | 477.459±456.834 | 97961 | 796.646±392.498 |
| HCT116 | 77630 | 405.224±237.451 | 65110 | 789.531±275.898 |

## Slide 4
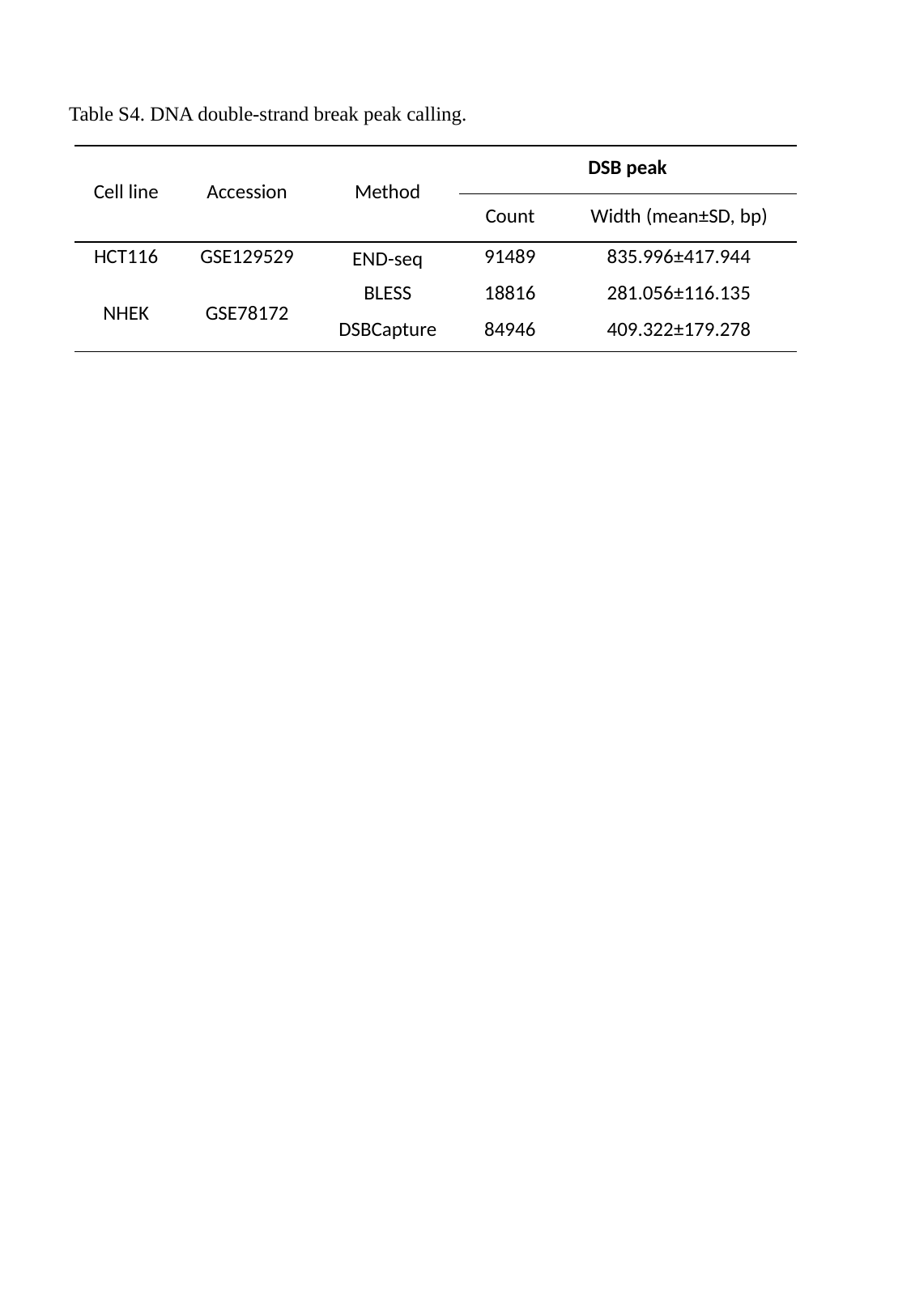

Table S4. DNA double-strand break peak calling.
| Cell line | Accession | Method | DSB peak | |
| --- | --- | --- | --- | --- |
| Cell line | | Accession | Count | Width (mean±SD, bp) |
| HCT116 | GSE129529 | END-seq | 91489 | 835.996±417.944 |
| NHEK | GSE78172 | BLESS | 18816 | 281.056±116.135 |
| | | DSBCapture | 84946 | 409.322±179.278 |
