# Supplementary material for: Stratifying TAD boundaries pinpoints focal genomic regions of regulation, damage, and repair
Source: Brief Bioinform. 2024 Jun 27;25(4):bbae306. doi: 10.1093/bib/bbae306 (PMC11210073; doi:10.1093/bib/bbae306)
Supplement: Supplementary_Figures_legend_bbae306 [file supplementary_figures_legend_bbae306.docx]

# Supplementary figure legend

Figure S1. TAD boundary identification at various resolutions.

(A) Statistics of TAD boundaries classified by level across a spectrum of resolutions, including 5kb, 10kb, 25kb, and 50kb, in seven distinct cell lines. (B) Barplot showing the fraction of high-level TAD boundaries (level3+) identified at 10kb resolution preserved at different resolution.

Figure S2. Relationship between gene characteristics and the level of the boundary at which they are located.

(A) Comparison of expression level for genes located at the boundary or not. (B) Distribution of gene expression levels for genes whose promoter (+/- 1kb from TSS) overlap with TAD boundaries. Genes are classified by the level of TAD boundaries. * *P* < 0.05, ** *P* < 0.01, *** *P* < 0.001, **** *P* < 0.0001, using Wilcoxon-test. (C) The mean expression level was calculated for each group of genes (level0-level3+) in the seven cell lines, showing a significant positive correlation. (D) The CRISPR scores (i.e., gene essentiality, with low values representing high degrees of essentiality) were lower for genes located at the boundary than for genes not located at the boundary. (E) The mean CRISPR score value was calculated for each group of genes (level0-level3+). (F) Comparison of the gene length. (G) Comparison of the number of transcript types for genes located at the boundary or not.

Figure S3. Recognition of TAD boundaries across reference methods.

(A) The barplot illustrates the fraction of boundaries called by the OnTAD method overlapping with those called by other reference methods. The number of methods is differentiated by color. (B) Comparison of different level boundaries recognizable by other methods.

Figure S4. Benchmarking analysis of the TAD boundaries that overlap in majority (>=3) of the reference approaches based on boundary classification.

(A-C) Comparison of characteristics for genes whose promoter (+/- 1kb from TSS) overlap with TAD boundaries, including expression level (A), CRISPR score (essentiality) of genes (B) and gene length (C). * *P* < 0.05, ** *P* < 0.01, *** *P* < 0.001, **** *P* < 0.0001, using Wilcoxon-test. (D-F) Comparison of CTCF enrichment between different grouped boundaries, both in terms of the fraction of boundaries overlapping with CTCF peaks (D, Fisher’s exact test), comparison of average number of overlapping CTCF sites on individual boundaries (E), and visualization of CTCF signal abundance at boundaries (F).

Figure S5. Functional genes tend to be enriched at high level boundaries.

(A-C) Barplots showing the enrichment of functional genes located at different level boundaries in HCT116 cell line. The fraction of genes located within each specific boundary is presented in the respective column. (D-E) Boxplots showing boundary level differences of essential genes or housekeeping genes. **** *P* < 0.0001, using Wilcoxon-test.

Figure S6. Exploration of TFBS-clustered regions located at boundaries and definition of boundary conservation score.

(A) The number of TFBS-clustered regions (right y-axis, black circles) and distribution of genomic annotation classes (left y-axis, colors) as a function of TFBS complexity (x-axis) in GM12878 cell line. (B) High complexity TFCRs are enriched at high level boundaries. (C) The averages boundary level of TFBS-clustered regions categorized by ascending complexity. (D) TFCRs in TC9 group (highest complexity) are located at a significantly higher boundary level. **** *P* < 0.0001, using Wilcoxon-test. (E) Upset plots show frequency distribution of boundaries across seven cell lines and an illustration of the Conservation Score. Conservation Score = 7 represents a high degree of conservation across seven cell lines, with lower scores indicating more specificity. (F) Mean conservation score was calculated for different level boundaries.

Figure S7. DSBs are not randomly distributed across the genome but rather exhibit genome bias.

(A-C) Barplots shows the fraction of genes with DSB peaks overlap in different groups (A, essential genes; B, housekeeping genes; C, old genes). DSBs are enriched at functional genes. **** *P* < 0.0001, using Fisher’s exact test. (D) Comparison of different levels for the mean number of DSBs per boundary. (E) Enrichments calculated from the DSB bigwig file within different level boundaries in HCT116 cell line. ANOVA test *p* value = 0.00000449. (F-G) Distribution of DSBs at boundaries of distinct categories. Boundaries were classified based on levels (F) or overlap with TFBS-clustered regions (G). Top: Average distribution of DSBs within ±50 kb of boundaries. Bottom: Heatmaps centered on boundaries. The color bar represents the intensity range based on DSB enrichment, with white to navy representing low to high enrichment values. (H) Mean values of containing DSB motifs for level3+ boundaries with TC9 overlapping, compared to level1/2 boundaries (gray) or level3+ boundaries without TC9 (yellow). *De novo* motif mining for the DSB peaks and the comparison of motif enrichment were performed using MEME Suite. Top motifs enriched in DSBs were determined according to the parameter *E*-value < 0.05.

Figure S8. Genome-wide DSB enrichment correlates with structural and epigenetic traits are determined across datasets.

(A) Boxplots comparing the DNase-seq signal of bins categorized by level. **** *P* < 0.0001, using Wilcoxon-test. (B) Heatmap showed comparison of DSB bigwig signal among genomic bins categorized by ascending DNase-seq signal and bin level. Related to Fig. 4A. (C-D) Comparison of genome-wide DSB enrichment in different datasets (DSBs called using two different methods in NHEK cell lines). In C, only the bins with gene overlapping were compared. (D) The number of bins categorized by number of DSBs.

Figure S9. Comparative analysis of bin based on DSB number classification.

(A) The number of bins categorized by number of DSBs. (B-C) Boxplots comparing the DNase-seq signal of bins categorized by number of DSBs and the expression level of genes located in each group in NHEK cell line. * *P* < 0.05, ** *P* < 0.01, **** *P* < 0.0001, using Wilcoxon-test.

Figure S10. High level of boundaries correlates with DNA repair.

(A) GO enrichment analysis for genes located at the high-level boundaries (level2+) in all seven cell lines (n=827) showed relevance to regulation of double-strand break repair after the boundaries were expanded to 3bin. (B) Barplots indicated repair-related genes (from Hussmann *et al.*, n=476) were significantly enriched at boundaries relative to all protein-coding genes. The plot showed the fraction of genes whose promoter (+/- 1kb from TSS) overlap with TAD boundaries in the gene lists. The comparison includes the case where the gene is located at the boundary in all seven cell lines and the case where the gene is at a high-level boundary in least one cell line. *** *P* < 0.001, **** *P* < 0.0001, using Fisher’s exact test. (C-D) Motif matches enriched at the high-level (level3+) with high-complexity TFBS-clustered region (TC9) boundaries in each cell line, related to Fig. 5F. The width of a single motif that MEME reports is set by the parameter “-maxw” (C: -maxw 15; D: -maxw 20). The motif enrichment *p*-value was derived from Tomtom tool in MEME Suite with default setting.

Figure S11. Representative example of “hub-boundary” and multi-omics landscape in GM12878 cell line.

(A) The captured region is chr3, 178.0–181.0 Mb near the *ACTL6A* gene. The upper panels show nested TAD structures. The high-level boundary (marked by yellow stripe), i.e., shared by multiple TADs, is accompanied with high gene expression, high chromatin accessibility and high-complexity TFBS-clustered regions (marked by elliptical circles).
